# Both Chromosome Decondensation and Condensation Are Dependent on DNA Replication in *C. elegans* Embryos

**DOI:** 10.1016/j.celrep.2015.06.046

**Published:** 2015-07-09

**Authors:** Remi Sonneville, Gillian Craig, Karim Labib, Anton Gartner, J. Julian Blow

**Affiliations:** 1 Centre for Gene Regulation and Expression, College of Life Sciences, University of Dundee, Dundee DD1 5EH, UK; 2 MRC Protein Phosphorylation and Ubiquitylation Unit, College of Life Sciences, University of Dundee, Dundee DD1 5EH, UK

## Abstract

During cell division, chromatin alternates between a condensed state to facilitate chromosome segregation and a decondensed form when DNA replicates. In most tissues, S phase and mitosis are separated by defined G1 and G2 gap phases, but early embryogenesis involves rapid oscillations between replication and mitosis. Using *Caenorhabditis elegans* embryos as a model system, we show that chromosome condensation and condensin II concentration on chromosomal axes require replicated DNA. In addition, we found that, during late telophase, replication initiates on condensed chromosomes and promotes the rapid decondensation of the chromatin. Upon replication initiation, the CDC-45-MCM-GINS (CMG) DNA helicase drives the release of condensin I complexes from chromatin and the activation or displacement of inactive MCM-2–7 complexes, which together with the nucleoporin MEL-28/ELYS tethers condensed chromatin to the nuclear envelope, thereby promoting chromatin decondensation. Our results show how, in an early embryo, the chromosome-condensation cycle is functionally linked with DNA replication.

## Highlights

• Replication initiation triggers the rapid decondensation of chromatids
• Replication initiation counteracts condensins, inactive MCM-2–7, and MEL-28
• DNA replication promotes condensin II accumulation and chromosome condensation

## Graphical Abstract

**Figure:**
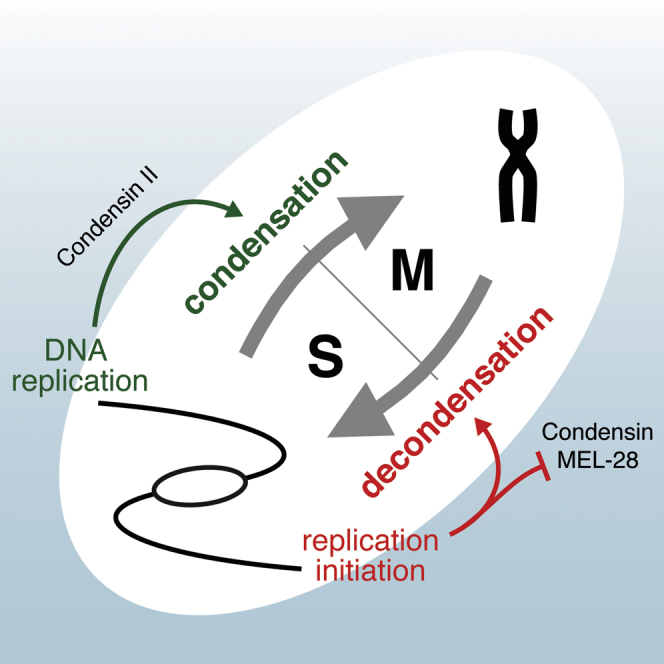

## Teaser

Chromatin organization changes dramatically within a cell cycle. Sonneville et al. find that in rapidly dividing *C. elegans* embryos, DNA replication initiation triggers rapid decondensation of chromatids. In addition, DNA replication promotes chromosome condensation in prophase. Therefore, the chromosome-condensation cycle and DNA replication are functionally linked.

## Introduction

Cell-cycle progression requires the ordered succession of cell-cycle stages, and checkpoints ensure that critical cell-cycle events such as DNA replication or chromosome alignment are completed before subsequent stages can occur. Changes in cyclin-dependent kinase (CDK) kinase activity and differential cyclin association drive major transitions such as the initiation of S phase, mitosis, and the subsequent segregation of chromatids. Faithful chromosome segregation requires the structural reorganization of chromosomes into condensed metaphase chromosomes, which is needed for the segregation of chromatids during anaphase. Conversely, chromosome decondensation facilitates transcription and DNA replication. In rapidly dividing embryos, S phase and mitosis alternate without apparent G1 or G2 phases. Thus, decondensation, DNA replication, and re-condensation occur in a short period and could potentially overlap. Indeed, we know little about how DNA replication and chromatin condensation and decondensation are coordinated.

Condensation is mediated by condensin complexes, pentameric ring-shaped structures composed of two structural maintenance of chromosomes (SMC) subunits that exhibit ATPase activity and that are related to cohesin subunits, plus three regulatory units known as chromosome associated proteins (CAPs). Most organisms contain two condensin complexes, condensin I and II, which share the same SMC units (MIX-1/SMC2 and SMC-4 in the worm) but differ in their regulatory subunits, termed CAPG-1, DPY-26, and DPY-28 for *C. elegans* condensin I and CAPG-2, KLE-2, and HCP-6 for *C. elegans* condensin II (Csankovszki et al., 2009; Hirano, 2012; Piazza et al., 2013; Thadani et al., 2012). In vertebrates, condensin I is cytoplasmic during interphase and appears to stabilize chromosome rigidity after nuclear envelope breakdown (Hirota et al., 2004; Ono et al., 2004). Condensin II is nuclear, is required for sister chromatid resolution during S phase, and promotes chromosomal axis formation during prophase (Cuvier and Hirano, 2003; Ono et al., 2013). In *C. elegans* embryos, condensin II is required for condensation during prophase and is concentrated on chromosomal axes (Csankovszki et al., 2009; Hagstrom et al., 2002; Kaitna et al., 2002). Worm condensin I is cytoplasmic, localizes to chromosomes after nuclear envelope breakdown, and appears to be required for chromosome segregation (Csankovszki et al., 2009). In *C. elegans*, a third condensin complex functions in dosage compensation for sex chromosomes (Meyer, 2010). Recent evidence suggests that condensin rings encircle DNA (Cuylen et al., 2011, 2013), and chromosome compaction might involve the entrapment of more than one DNA molecule or the interaction of condensin rings (for review, see Thadani et al., 2012).

Eukaryotic DNA replication is divided into two non-overlapping phases (Blow and Dutta, 2005; DePamphilis et al., 2006). In late mitosis and early G1, replication origins are licensed for replication by loading Mcm2–7 double hexamers, which requires the loading factors ORC, Cdc6, and Cdt1. During S phase, CDKs and Dbf4-dependent kinases activate the Mcm2‐7 helicase by promoting its interaction with Cdc45 and the GINS complex (Gambus et al., 2006; Ilves et al., 2010; Moyer et al., 2006). This CMG (Cdc45-MCM-GINS) helicase unwinds the template DNA, allowing for RPA (single-strand binding protein) binding and DNA synthesis by DNA polymerases. In *C. elegans* embryos, chromatin is licensed at the end of M phase; when nuclei form and chromatin decondenses, licensing factors are then exported from nuclei, thereby ending the licensing phase and preventing re-replication (Sonneville et al., 2012). The rapidly dividing early embryo has relatively weak cell-cycle checkpoints, which allow continued cycling even when essential processes, such as DNA replication, are defective (Brauchle et al., 2003; Budirahardja and Gönczy, 2009; Encalada et al., 2000). Cell-cycle analysis is further facilitated by the rapid turnover of cytosolic proteins in the gonad (Oegema and Hyman, 2006) and the extended period of time needed for progressing from the premeiotic S phase through the extended meiotic prophase (Jaramillo-Lambert et al., 2007), which allows depletion of replication genes before the first embryonic S phase without affecting the previous, pre-meiotic S phase (see below).

Here, we investigate the functional relationship between DNA replication and the chromosome-condensation cycle by combining RNAi and in vivo imaging in *C. elegans* embryos. We show that replication commences concomitant with decondensation and that replication initiation, but not elongation, promotes decondensation. We provide evidence that replication initiation is needed for the dissociation of the MEL-28 (ELYS) nucleoporin from chromatin during chromosome decondensation and that MEL-28 depletion rescues the decondensation defect associated with blocking replication initiation. Finally, we show that genome duplication is required for condensin II concentration on chromosomal axes and for proper chromatin condensation in prophase. Our results reveal that DNA replication and the chromosome-condensation cycles are tightly coupled.

## Results

**Figure 1:**
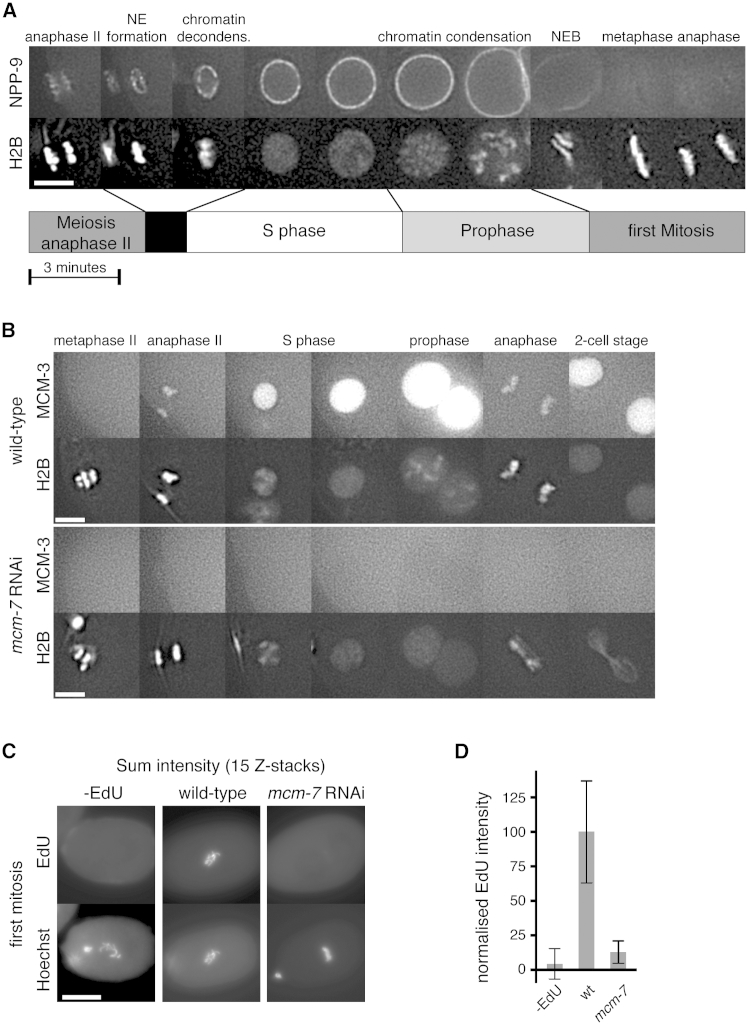
*C. elegans* Chromatin Dynamics (A) Chromatin dynamics in relation to the nuclear envelope cycle. Images taken from a time-lapse sequence of an embryo expressing GFP-NPP-9 (top row) and mCherry-H2B (bottom row) progressing from the second meiotic division to the first embryonic cell cycle. The timeline below indicates the duration of cell-cycle stages. (B) Chromatin dynamics and MCM-2–7. Images from wild-type and *mcm-7* RNAi embryos expressing GFP-MCM-3 (top rows) and mCherry-H2B (bottom rows). (C) EdU incorporation during the first embryonic S phase. Permeabilized embryos pulsed with EdU from meiosis II to the first embryonic mitosis. EdU uptake (top images) and DNA (bottom images) determined during the first mitosis. (D) Quantification of EdU incorporation for ten embryos. Error bars represent SD. Scale bars represent 5 μm (A and B) and 20 μm (C). See Movies S1 and S2 for embryos shown in (A) and (B).

We used RNAi and in vivo imaging of *C. elegans* embryos to uncover the links between DNA replication and the chromosome-condensation cycle. We visualized chromosomal DNA by employing mCherry fused to histone 2B (H2B) (mCherry-H2B) and the nuclear envelope by using the nucleoporin NPP-9 fused to GFP. Figure 1A and Movie S1, where the entire embryo is displayed, show the sequence of events occurring from meiotic anaphase II to the end of the first embryonic cell cycle. Shortly after fertilization, oocyte-derived chromosomes, which we will refer to as female chromosomes, complete the two meiotic divisions (meiosis I and II) leading to the extrusion of the two polar bodies and the formation of a haploid female nucleus. At the end of anaphase II, a ring of GFP-NPP-9 forms around the female chromosomes, followed by rapid decondensation of the chromatin. The female and male haploid nuclei grow in size at opposite poles of the embryo concomitant with the bulk of DNA replication (Edgar and McGhee, 1988). These nuclei then migrate toward each other and meet at the posterior half of the cell and then move to the center of the cell. Concomitant with nuclear migration, chromatin patches indicative of condensation form and distinct chromosomes become progressively apparent. Upon nuclear disassembly, indicated by the disappearance of nucleoporins, chromosomes congress on the metaphase plate and anaphase ensues (Figure 1A; Movie S1).

### Chromosome Condensation Occurs Abnormally if DNA Replication Is Inhibited

**Figure 2:**
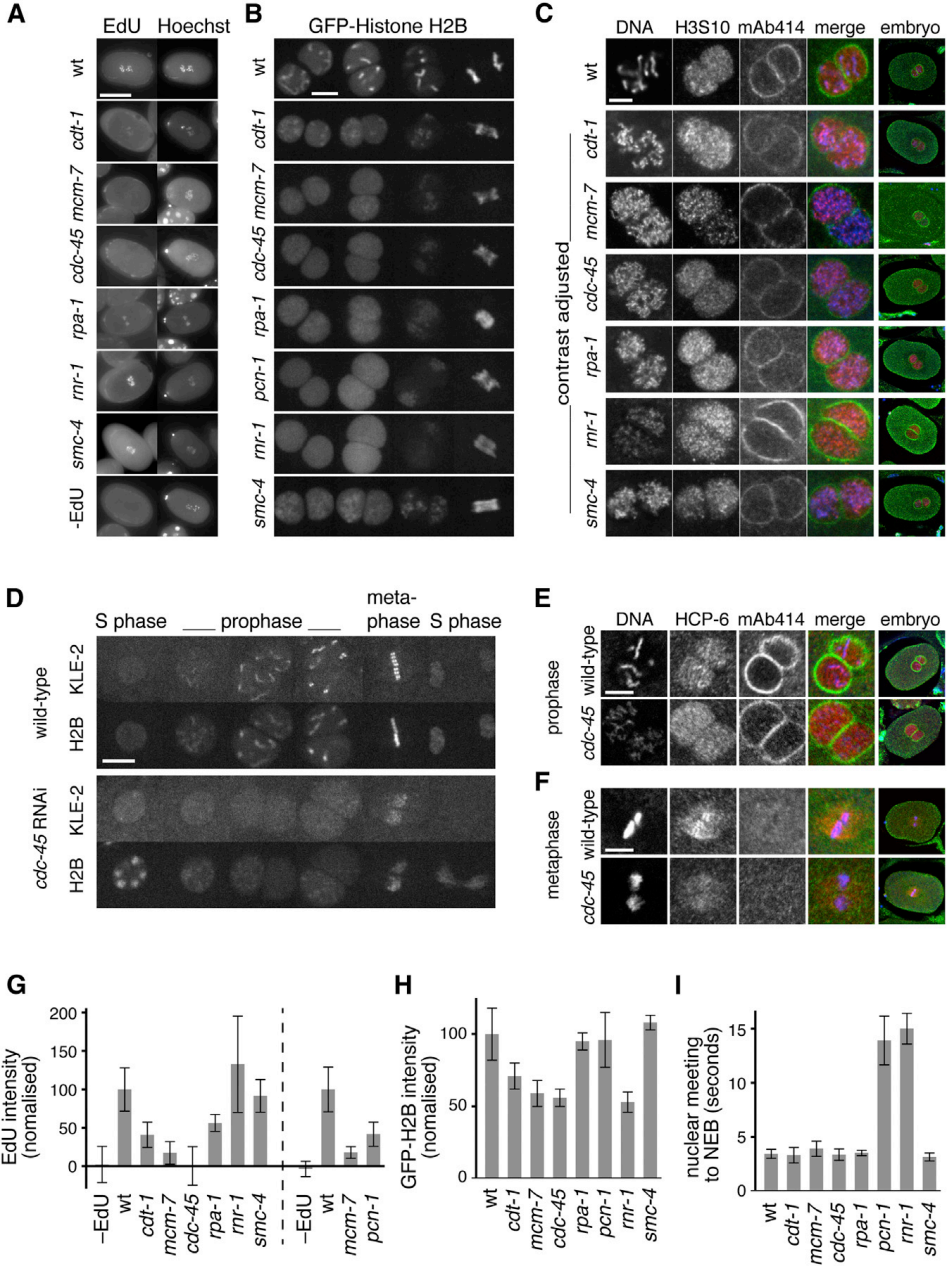
Chromatin Condensation Requires Duplicated DNA (A and G) EdU incorporation assayed during the first S phase. (A) Permeabilized embryos pulsed with EdU for 30 min and stained at mitosis for EdU (left images) and DNA (right images, Hoechst). (B) Visualization of chromosome condensation during the first mitosis. Images taken from time-lapse sequences of wild-type, *cdt-1*, *mcm-7*, *cdc-45*, *rpa-1*, *pcn-1*, *rnr-1*, and *smc-4* RNAi embryos expressing GFP-H2B. (C) Visualization of chromosome condensation during prophase. Wild-type, *cdt-1*, *mcm-7*, *cdc-45*, *rpa-1*, *rnr-1*, and *smc-4* RNAi embryos stained for phospho-H3S10 (red), nuclear pores (mAb414, green), and DNA (blue). Chromatin imaged with a higher magnification is shown on the left. (D) Visualization of the condensin II subunit KLE-2. Images taken from time-lapse sequences of wild-type and *cdc-45* RNAi embryos expressing GFP-KLE-2 (top images) and mCherry-H2B (bottom images). (E and F) Visualization of HCP-6. Wild-type and *cdc-45* RNAi embryos stained for HCP-6 (red), nuclear pores (mAb414 green) and DNA (blue). Magnified images of nuclei are shown on the left. (E) Late prophase. (F) Metaphase. (G) Quantification of EdU incorporation for ten embryos prepared as in (A). (H) Quantification of GFP-H2B on chromatin during mitosis from five embryos prepared as in (B). (I) Duration between the meeting of male and female nuclei and nuclear envelope breakdown (NEB) from five embryos prepared as in (B). Scale bars represent 20 μm (A) and 5 μm (B–E). Error bars represent SD. See Movies S3 and S4 for embryos shown in (B) and in (D).

Within the gonad of an adult worm, the differentiation of a mature oocyte from a mitotic germ cell takes >24 hr (Jaramillo-Lambert et al., 2007), a period of time sufficient to inactivate genes by RNAi in the embryo without affecting the premeiotic S phase. As a first step to assess the relationship between DNA replication and chromosome condensation, we used RNAi to deplete the licensing factor MCM-7. The assembly of hexameric MCM-2–7 onto chromosomes during anaphase requires the presence of all six MCM-2–7 subunits. We therefore used GFP-MCM-3 as a marker for MCM-2–7 chromatin loading. When MCM-7 was depleted, GFP-MCM-3 failed to load onto anaphase chromosomes or accumulate in nuclei during S phase, consistent with the inactivation of the MCM-2–7 complex (Sonneville et al., 2012) (Figure 1B). When *mcm-7* was knocked down by RNAi, chromosome condensation was compromised during prophase of the first embryonic cell cycle and massive chromatin bridges were observed during anaphase (“cut phenotype”) (Figure 1B; Movie S2). In addition, we observed a delay in chromosome decondensation at the end of meiotic anaphase II (Figure 1B, S phase; Movie S2), a phenotype we will examine in more detail below. Such a cut phenotype has been associated with replication defects in fission yeast and human cells (Hirano et al., 1986; Samejima et al., 1993; Steigemann et al., 2009) and is thought to be a consequence of cells passing through mitosis with unreplicated DNA. As an aside, in MCM-7-depleted embryos, no chromatin bridges were observed during the meiotic anaphases (Figure 1B, anaphase II), suggesting that the pre-meiotic S phase was not affected. In order to show directly that MCM-7 depletion inhibits replication, we adapted 5-ethynyl-2′-deoxyuridine (EdU) labeling procedures to measure replication in the entire first embryonic cell cycle (see Experimental Procedures). Permeabilized embryos incubated with EdU throughout the first embryonic cell cycle showed a ∼90% reduction of EdU incorporation after one cell cycle when MCM-7 was depleted (Figures 1C and 1D). In agreement with compromised EdU incorporation, GFP-histone H2B intensity during the first mitosis of *mcm-7* RNAi embryos was approximately half of wild-type, consistent with an almost complete block in replication during the first embryonic S phase (Figures 2B and 2H). Therefore, during the first embryonic cell cycle, MCM-2–7 are required for replication, condensation, and segregation of the chromatin.

We next wished to test if other replication genes are required for chromatin condensation. The majority of *C. elegans* genes have been systematically depleted by RNAi, and DIC (differential interference contrast) recordings of the first embryonic cell cycles have been deposited into “phenobank” (Sönnichsen et al., 2005). We screened phenobank to identify replication genes whose inactivation led to a cut phenotype akin to MCM depletion. Examining knockout phenotypes of 40 genes expected to be involved in DNA replication, we found that 14 were associated with a first-cycle cut phenotype (Table S1). The absence of a cut phenotype in other replication mutants is consistent with carryover of maternal protein, partial RNAi depletion, or genetic redundancy. To further analyze regulatory connections between DNA replication and the chromosome condensation cycle, we focused on replication factors involved in various stages of replication whose depletion gave a first-cycle cut phenotype. We thus depleted these replication factors: the CDT-1 licensing factor, MCM-7, the CDC-45 initiation factor, the RPA-1 single-strand binding protein, the proliferating cell nuclear antigen (PCNA) ortholog PCN-1, and the RNR-1 ribonucleotide reductase required for dNTP supply. With the exception of *rnr-1*, each of these RNAi treatments caused a large reduction in EdU incorporation (Figures 2A and 2G). The EdU incorporation in embryos treated with *rnr-1* RNAi can be explained by the depletion of the cellular dNTP pools, which favors the incorporation of EdU (itself not requiring ribonucleotide reductase) during residual replication. In addition, with the exception of *rpa-1 and pcn-1*, each of these RNAi treatments halved the intensity of chromatin bound GFP-H2B at first mitosis, consistent with a replication block (Figures 2B and 2H). The high GFP-H2B intensity in cells treated with *rpa-1 and pcn-1* RNAi is surprising, but it may reflect an abnormal chromatin structure in cells lacking these factors. Recordings of GFP-H2B show that all of these depletions led to defective chromosome condensation at first mitosis (Figure 2B; Movie S3). Analysis of Hoechst-stained chromosomes (Figure 2C, left panels) of late prophase embryos, as defined by the position of the nuclei and by staining for phosphorylated Ser10 of histone H3 (Figure 2C), indicated that condensation was not completely abolished; chromatin patches formed during prophase but did not congress into single chromosomes. Such patches were much less discernible when GFP-H2B was imaged, likely due to the background of nucleoplasmic GFP-H2B. Defective condensation was followed by massive anaphase bridging (Figure 2B; Movie S3). Such condensation and segregation defects resemble the effect of inactivating the condensin II complex (Csankovszki et al., 2009) (Figure 2B, *smc-4*; Movie S3; see below). We observed that nuclear accumulation of GFP-AIR-2 (the *C. elegans* Aurora B homolog) and the commencement of chromosome condensation occur at the same time in wild-type embryos (Figure S1A). We thus asked if those two events are linked. GFP-AIR-2 nuclear accumulation occurred upon depletion of replication genes (Figures S1A and S1B, red arrows). Thus, assuming that GFP-AIR-2 and phospho-H3S10 staining serve as prophase makers, our data show that the condensation defect associated with the depletion of replication genes is not due to a lack of prophase (Figure S1A; Figure 2C).

We next investigated if S phase checkpoint activation is linked to chromosome-condensation defects. As in other organisms, the inhibition of replication fork elongation in *C. elegans* embryos leads to the activation of a cell-cycle checkpoint delaying entry into mitosis (Brauchle et al., 2003; Encalada et al., 2000; Korzelius et al., 2011). Consistent with earlier reports, inactivation of *pcn-1* and *rnr-1* caused a delay in nuclear envelope breakdown (Figure 2I) and *rnr-1* inactivation also caused a delay in the nuclear localization of GFP-AIR-2 (Figures S1A and S1C). In contrast, *cdt-1*, *mcm-7*, and *cdc45* RNAi embryos revealed little or no delay in cell-cycle progression and GFP-AIR-2 nuclear entry (Figure 2I; Figure S1C; Movies S3). These findings are consistent with a lack of replication fork initiation to such an extent that the replication checkpoint, which requires the generation of RPA-coated single-stranded DNA, cannot be activated (Zou and Elledge, 2003). In line with the expected role of RPA-1 in checkpoint activation, the depletion of RPA-1 also failed to elicit a cell-cycle delay but nevertheless lead to a defect in chromosome condensation (Figures 2B, 2C, and 2I).

**Figure 3:**
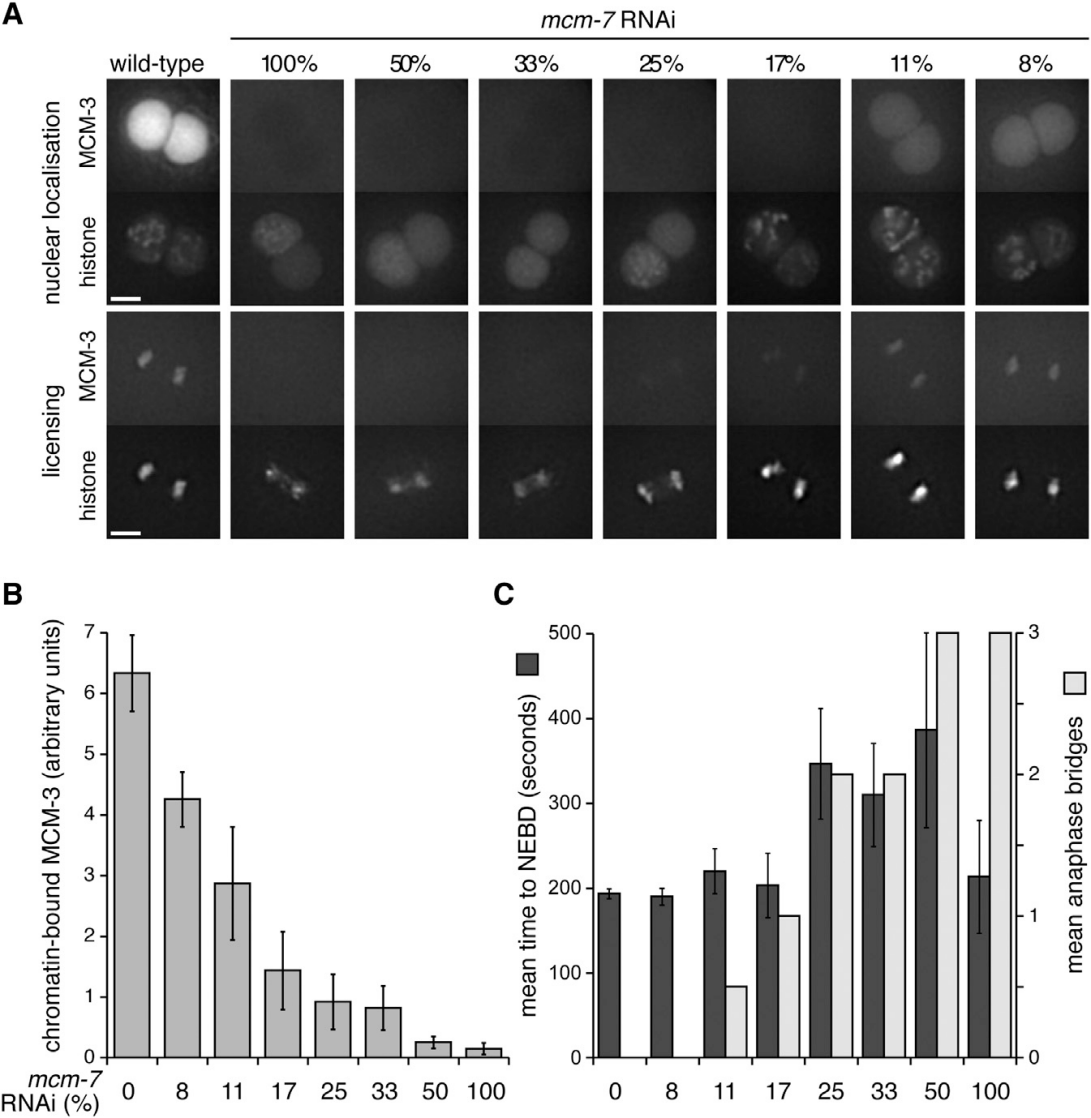
Graded Depletion of Chromatin Bound MCM-2–7 (A) Images taken from time-lapse sequences upon *mcm-7* RNAi titration in embryos expressing GFP-MCM-3 (upper images) and mCherry-H2B (lower images) during prophase (upper panel) and anaphase of mitosis (lower panel). The percentage of *mcm-7* RNAi expressing bacteria relative to bacteria containing an empty vector is shown. (B) Quantification of average chromatin bound GFP-MCM-3 1 min after anaphase onset (n = 3). (C) Average time elapsing between the meeting of nuclei and NEB (black bars) and the number of mitoses with anaphase bridges (white bars) (n = 3). Stronger staining of the mCherry-H2B reporter in the female chromatin as compared to the male chromatin, allowed to separately score anaphase bridges on female and male chromatins. Embryos with anaphase bridges on female and male chromatin scored 1, while embryos with anaphase bridges only on male chromatin scored 0.5. Scale bars are 5 μm. Error bars represent SD.

To substantiate further this interpretation, we employed a graded depletion of MCM-7 (Figure 3A). The maximum RNAi dose (100%) led to an undetectable level of MCM-3 loaded onto anaphase chromosomes (Figures 3A and 3B) and a failure to import GFP-MCM-3 into interphase nuclei. At this 100% RNAi dose, little or no delay in nuclear envelope breakdown was observed (Figure 3C, dark bars), indicating that there was no significant activation of the replication checkpoint. Consistent with our previous results, this high RNAi dose caused a failure of chromosome condensation and the formation of anaphase bridges (Figures 3A and 3C, light bars). Lower levels of *mcm-7* RNAi allowed a graded loading of GFP-MCM-3 onto chromatin (Figure 3B). RNAi doses ranging from 50% to 25% led to strong reduction of GFP-MCM-3 loading, the formation of anaphase bridges, and the maximal level of checkpoint activation as judged by lengthening of the cell cycle. This phenotype is expected if the number of chromatin-bound MCM-2–7 is insufficient to complete replication, while compromised replication triggers checkpoint activation. Limiting RNAi doses from 17% to 8% led to a less severe reduction of GFP-MCM-3 loading without checkpoint activation and without strong chromosome-condensation defects but with occasional anaphase bridges. This phenotype is consistent with the idea that an excess of MCM complexes are loaded onto “dormant” replication origins, which are not normally needed for genome duplication (Ge et al., 2007; Newman et al., 2013; Woodward et al., 2006).

It is established that condensin II is required for chromosome condensation during prophase (Hirano, 2012; Piazza et al., 2013; Thadani et al., 2012). We therefore generated worms expressing the GFP-KLE-2 condensin II subunit. As expected from previous studies, GFP-KLE-2 was diffused throughout the nucleoplasm during S phase and concentrated on chromosomal axes during prophase, each of the chromatid axes being resolved during metaphase (Figure 2D; Movie S4). Similarly, embryos stained for another condensin II subunit, HCP-6, showed chromatin-bound HCP-6 during prophase and metaphase (Figures 2E and 2F). In contrast, inactivation of *mcm-7*, *cdc-45*, or *rpa-1* prevented KLE-2 or HCP-6 foci formation during prophase (Figures 2D and 2E; Movie S4). Controls showed that KLE-2 nuclear localization required HCP-6 and that KLE-2 chromatin loading was dependent on the SMC-4 and HCP-6 condensin subunits (Figure S1D), while KLE-2 and HCP-6 were chromatin bound during mitosis when replication was blocked (Figures 2D and 2F; Movie S4). Taken together, our results suggest that condensin II concentration on chromosomal axes and chromosome condensation are both dependent on DNA having been replicated. Conversely, we found that blocking chromosome condensation by inactivating *smc-4* did not inhibit chromosome duplication, as measured by EdU incorporation (Figures 2A and 2G), GFP-CDC-45 binding to chromatin (data not shown), or duplication of chromatin-bound histones (Figure 2H).

### Activation of the CMG Helicase Promotes Chromatin Decondensation

**Figure 4:**
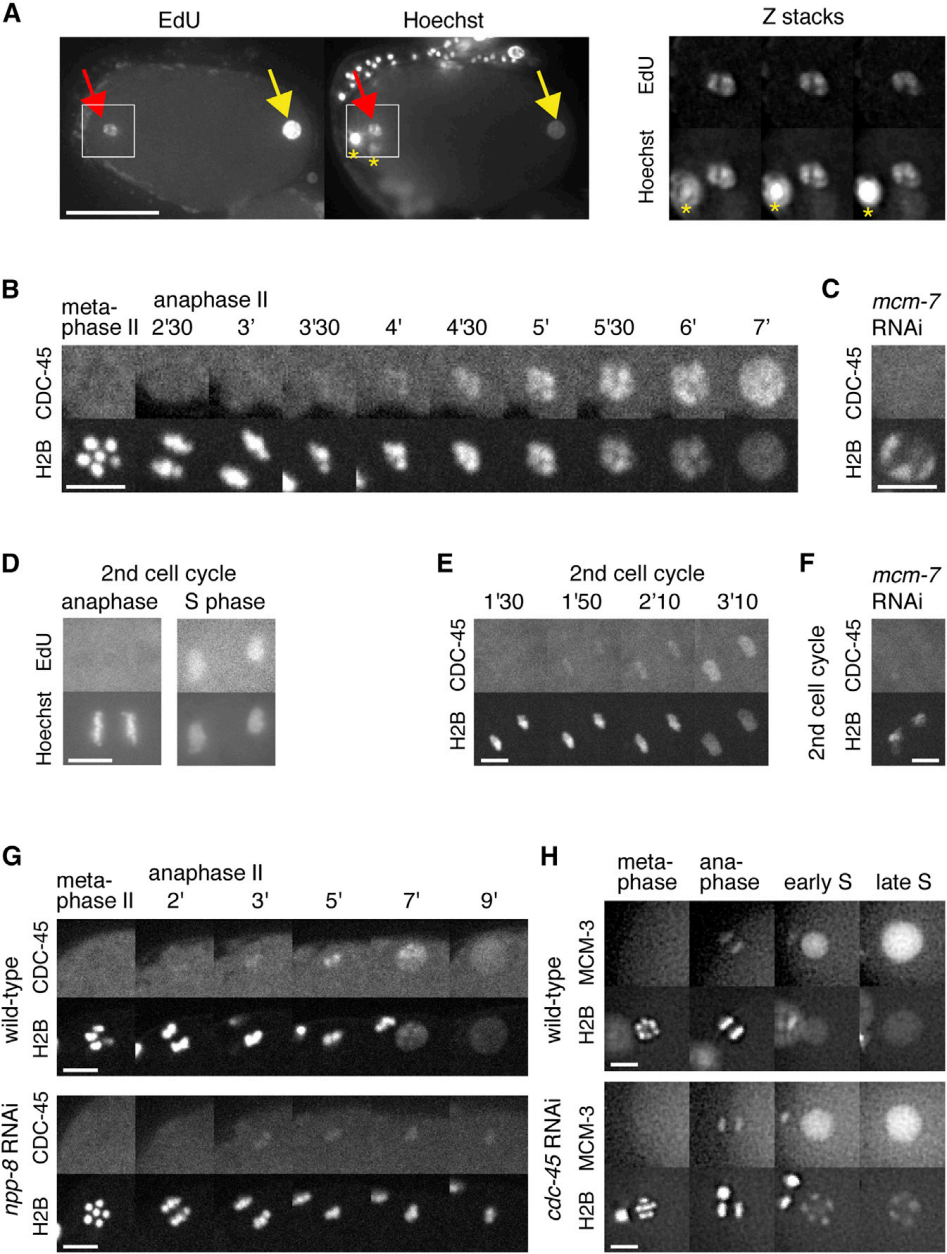
DNA Replication Initiates on Condensed Chromosomes (A) Timing of DNA replication initiation. Projections of 15 z sections spaced by 0.5 μm (left panel) showing EdU (left image) and DNA (Hoechst, right image). Permeabilized embryos pulsed for 10 min with EdU were stained. Red arrow indicates the female nucleus. Yellow arrow indicated the male nucleus. Yellow asterisks indicate polar bodies. Magnification of three Z-sections (right panel) showing EdU (top images) and DNA (bottom images) of the female nucleus. (B and C) GFP-CDC-45 binds chromatin during decondensation. Images taken from time-lapse sequences of embryos expressing GFP-CDC-45 (upper images) and mCherry-H2B (lower images) during chromatin decondensation of the female nucleus. (B) Wild-type. (C) *mcm-7* RNAi. (D) Permeabilized embryos pulsed for 5 min with EdU and stained during anaphase of the first mitosis (left panel) and S phase of the second cell cycle (right panel). Top images are EdU, and bottom images are Hoechst. (E and F) Images taken from time-lapse sequences of embryos expressing GFP-CDC-45 (upper images) and mCherry-H2B (lower images) during chromatin decondensation at the second cell cycle. (E) Wild-type. (F) *mcm-7* RNAi. (G) GFP-CDC-45 chromatin binding requires nuclear envelope formation. Images of wild-type (upper panel) and *npp-8* RNAi (lower panel) embryos expressing GFP-CDC-45 (upper images) and mCherry-H2B (lower images) during meiosis II and S phase. (H) Chromatin decondensation requires CDC-45. Images of wild-type (upper panel) and *cdc-45* RNAi (lower panel) embryos expressing GFP-MCM-3 (upper images) and mCherry-H2B (lower images) during meiosis II and S phase. Scale bars represent 20 μm (A) and 5 μm (B–H). Time is expressed in minutes and seconds relative to anaphase onset.

Having shown that DNA replication is required for chromosome condensation during prophase, we next wanted to address the converse question, namely, whether DNA replication is required to decondense the chromatin on exit from M phase. In cell types with a significant G1 phase, chromosome decondensation occurs well before DNA replication, but in certain embryonic cells (such as *Xenopus*, *Drosophila*, and *C. elegans* early embryos), which have either short G1 phases or lack them entirely, it is possible that chromosome decondensation and the initiation of DNA replication occur at the same time. In order to examine precisely when S phase starts relative to chromosome decondensation, we used EdU incorporation to label *C. elegans* embryos during early stages of S phase in the first cell cycle after fertilization. EdU staining in the female haploid nucleus was first observed in embryos with partially condensed DNA, indicating that some DNA replication occurs during decondensation (Figure 4A, left panel red arrow, right panels for magnification). As an aside, EdU staining at this stage was always stronger in the male nucleus, indicating that replication initiates earlier or occurs faster in the male nucleus (Figure 4A, yellow arrow). To corroborate that replication commences concomitant with decondensation, we investigated GFP-CDC-45 chromatin recruitment, which requires MCM-2–7 (Figures 4C and 4F) and serves as a marker for ongoing DNA replication (Sonneville et al., 2012). We found that GFP-CDC-45 localized to condensed chromatin 3–4 min after the onset of anaphase II, coinciding with nuclear envelope assembly. GFP-CDC-45 accumulated on chromatin as chromosomes decondensed (Figure 4B). To test if this also applies to other cell cycles, embryos passing though the second cell cycle were treated with a short (5 min) pulse of EdU, and we observed incorporation during decondensation, but not during anaphase (Figure 4D). GFP-CDC-45 also localized to chromatin during decondensation at this stage (Figure 4E), and its nuclear enrichment was largely dependent on MCM-2–7 (Figure 4F). Therefore, both EdU labeling and GFP-CDC-45 localization indicate that DNA replication starts on condensed chromosomes and continues during chromosome decondensation in at least two S phases following fertilization. The nucleoporin NPP-8/NUP155 is essential for both nuclear pore formation and chromatin decondensation (Galy et al., 2003; Sonneville et al., 2012). In embryos treated with RNAi against *npp-8*, the chromatin remained condensed and GFP-CDC-45 was reduced on chromatin, suggesting that DNA replication did not initiate properly (Figure 4G). This is consistent with the idea that nuclear assembly precedes chromatin decondensation and is required for the initiation of DNA synthesis in *C. elegans* embryos, as it is in *Xenopus* embryos (Blow and Sleeman, 1990; Blow and Watson, 1987; Newport, 1987).

**Figure 5:**
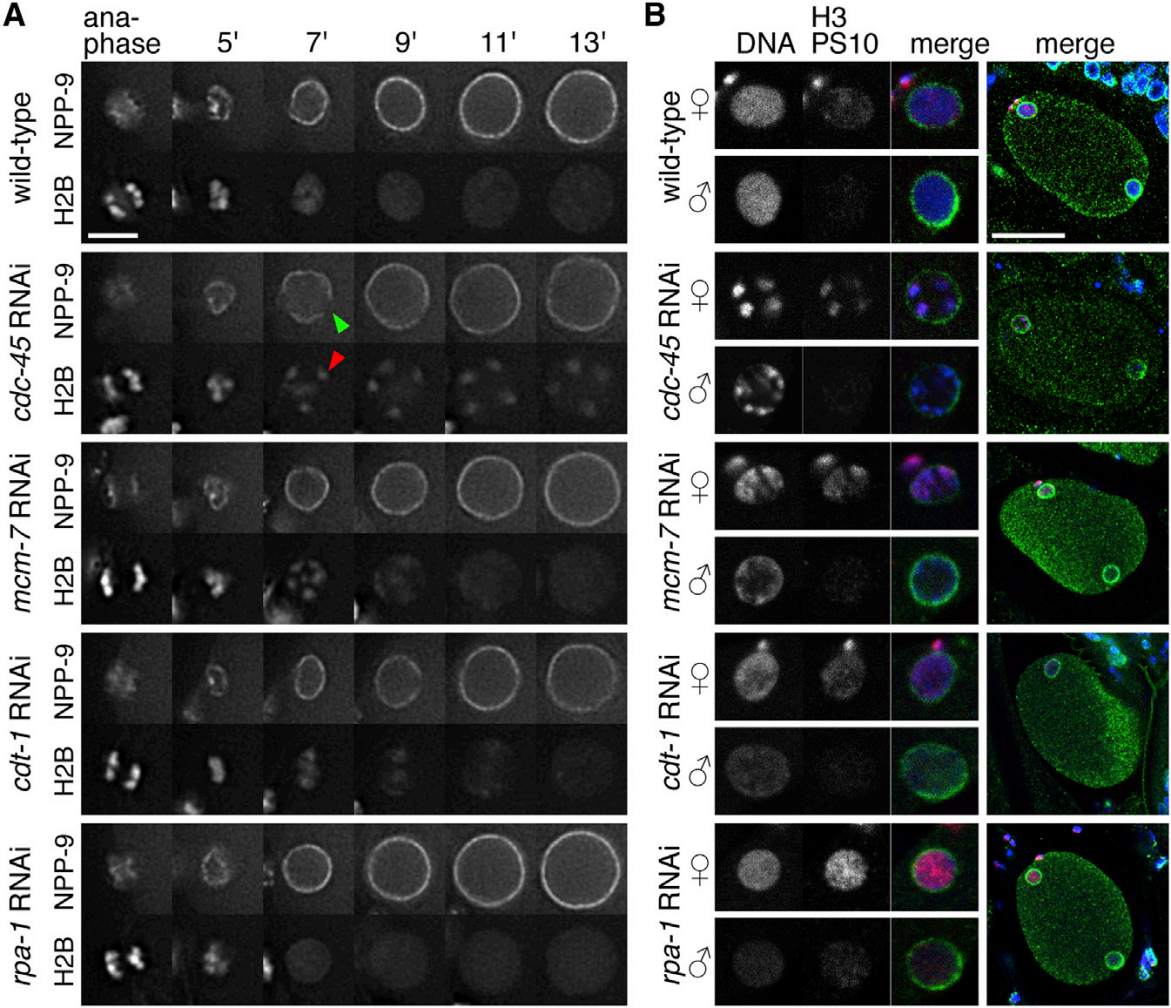
CMG Helicase Promotes the Rapid Chromatin Decondensation (A) Images taken from time-lapse sequences of wild-type, *cdc-45*, *mcm-7*, *cdt-1*, and *rpa-1* RNAi embryos expressing GFP-NPP-9 (upper images) and mCherry-H2B (lower images) during the first embryonic S phase. (B) Images of wild-type, *cdc-45*, *mcm-7*, *cdt-1*, and *rpa-1* RNAi embryos during early S phase. Embryos were stained for phospho-H3S10 (red), nuclear pore (mAb414, green), and DNA (blue). Magnifications of the female and male nuclei are shown on the left. Scale bars represent 5 μm (A) and 20 μm (B). See Movie S5 for embryos shown in (A).

We next explored whether DNA replication is required for chromosome decondensation. In *cdc-45* RNAi embryos, MCM-2–7 was loaded normally onto chromatin (Figure 4H, anaphase), but loading of GFP-RPA-1 was abolished at the least in the early stage of S phase (Figures S2A and S2B, 5 and 5.5 min, arrow), consistent with the expected role of CDC-45 in DNA unwinding. Strikingly, chromatids remained condensed at the nuclear periphery under such conditions, while nuclei increased in size (Figure 4H). The nuclear envelope was functional in *cdc-45* RNAi embryos, as nuclear accumulation of GFP-MCM-3 and GFP-RPA-1 occurred normally (Figure 4H; Figures S2A and S2D). When origin licensing was inhibited using RNAi against *mcm-7*, a mild phenotype was observed: chromosome decondensation was delayed relative to wild-type but occurred faster than in *cdc-45* RNAi embryos (Figures 5A and 5B). The phenotype of *cdt-1* depletion varied between embryos ranging from mild (three out of five embryos) to strong (two out of five) decondensation defects (Figure 5A; Movie S5; data not shown). In all cases, there was no change in the timing of GFP-NPP-9 recruitment. However, the initial NPP-9 distribution at times appeared uneven in *cdc-45* RNAi embryos (green arrowhead). Under all these conditions, early S phase chromatin had similar levels of phospho-H3S10 as compared to the wild-type (Figure 5B). In contrast, decondensation was normal when replication elongation was blocked by RNAi against *rpa-1* (Figures 5A and 5B), *rnr-1* or *pcn-1* (R.S., unpublished data). These findings indicate that chromosome decondensation requires activation of the CMG helicase during the initiation of chromosome replication but is independent of ongoing DNA synthesis during elongation.

**Figure 6:**
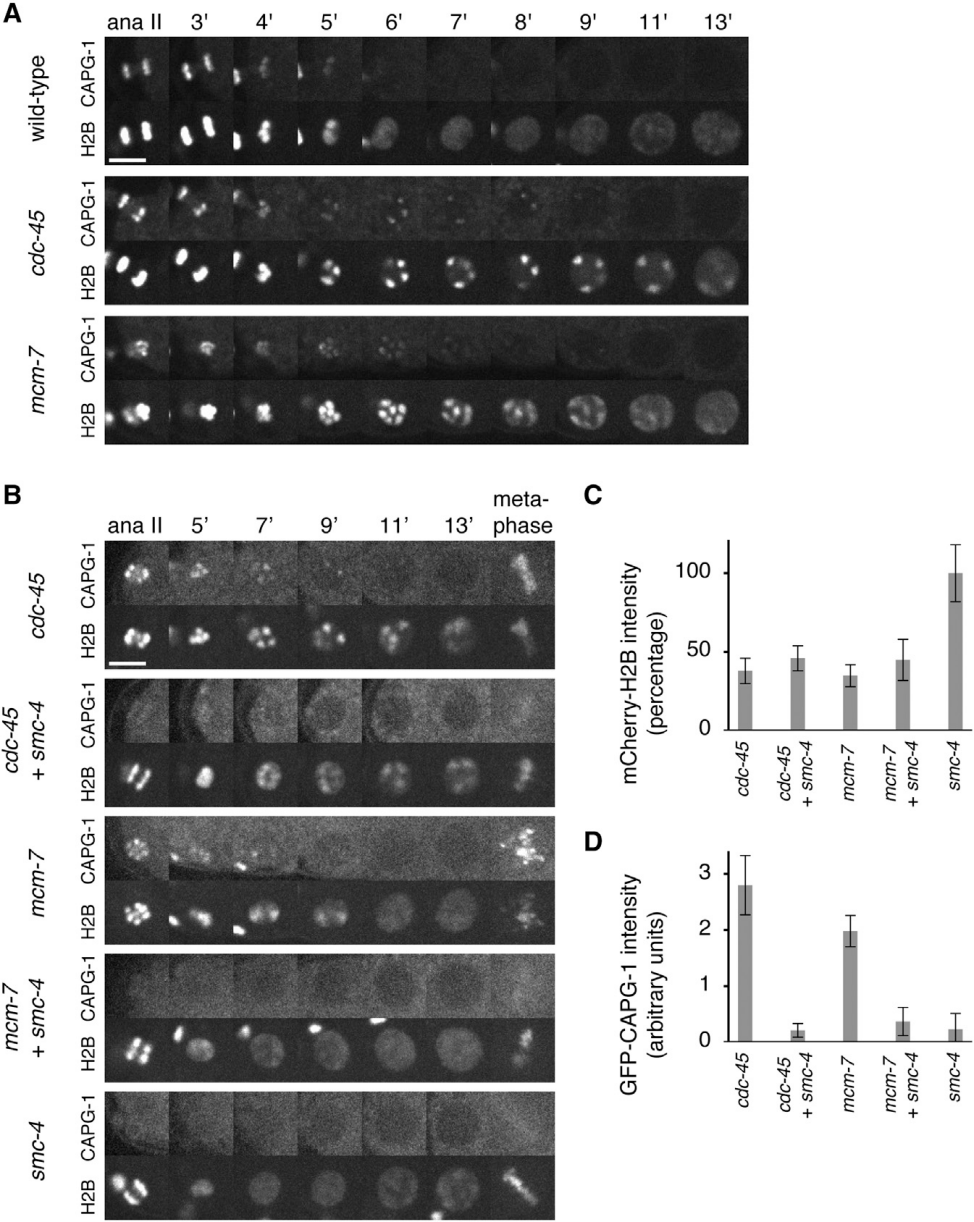
CMG Helicase Triggers Condensin Release from Chromatin (A) Images from wild-type, *cdc-45*, and *mcm-7* RNAi embryos expressing GFP-CAPG-1 (top images) and mCherry-H2B (bottom images) during the first embryonic S phase. (B) Images from *cdc-45* single, *cdc-45*; *smc-4* double, *mcm-7* single, *mcm-7*; *smc-4* double and *smc-4* single RNAi embryos expressing GFP-CAPG-1 (top images) and mCherry-H2B (bottom images) during the first embryonic S phase. (C and D) Quantification of mCherry-H2B (C) or GFP-CAPG-1 (D) chromatin enrichment at the first mitosis averaged from five embryos. Error bars represent SD. See Movie S6 for embryos shown in (B).

To explore the mechanism by which replication initiation drives chromatin decondensation, we first analyzed the CAPG-1 component of condensin I, which associates with condensed chromosomes during mitosis and is excluded from nuclei during interphase (Csankovszki et al., 2009; Hirano, 2005; Hudson et al., 2009). Figure 6A shows that GFP-CAPG-1 (Collette et al., 2011) was released rapidly from nuclei in wild-type embryos exiting from meiosis but persisted on condensed nuclear chromatin after RNAi to *cdc-45* or *mcm-7*. These results show that the observed decondensation defects correlate with persistent chromatin binding of condensin I.

To test whether condensin is required to maintain chromatin condensation when the initiation of replication is blocked, we performed double depletions of *mcm-7* and *smc-4*, or *cdc-45* and *smc-4*, in embryos expressing GFP-CAPG-1 and mCherry-H2B. Quantitation of mCherry-H2B intensity after one cell cycle showed that the level of H2B was halved upon both double depletions, as compared to the single *smc-4* inactivation, consistent with a complete lack of replication following depletion of *mcm-7* or *cdc-45* (Figure 6C). Moreover, the chromatin binding of GFP-CAPG-1 during metaphase was strongly reduced in all embryos treated with *smc-4* RNAi (Figures 6B and 6D). As predicted by the removal of condensin from chromatin, decondensation was restored in embryos treated with *mcm-7 smc-4* double RNAi, equivalent to the *smc-4* single treatment (Figure 6B; Movie S6). Interestingly, however, double depletion of *cdc-45* and *smc-4* only led to the partial restoration of chromatin decondensation upon entry into S phase. Chromatin was more diffuse in early interphase nuclei of *cdc-45 smc-4* embryos, compared to single *cdc-45* RNAi embryos (Figure 6B, compare nuclei at 7 and 9 min after anaphase onset; Movie S6), but chromatin patches were still observed and they persisted throughout S phase. Therefore, although the decondensation defect of *mcm-7* RNAi embryos can be explained by the persistence of condensin on chromatin, these findings suggest that an additional condensin-independent mechanism impairs decondensation in *cdc-45* RNAi embryos.

**Figure 7:**
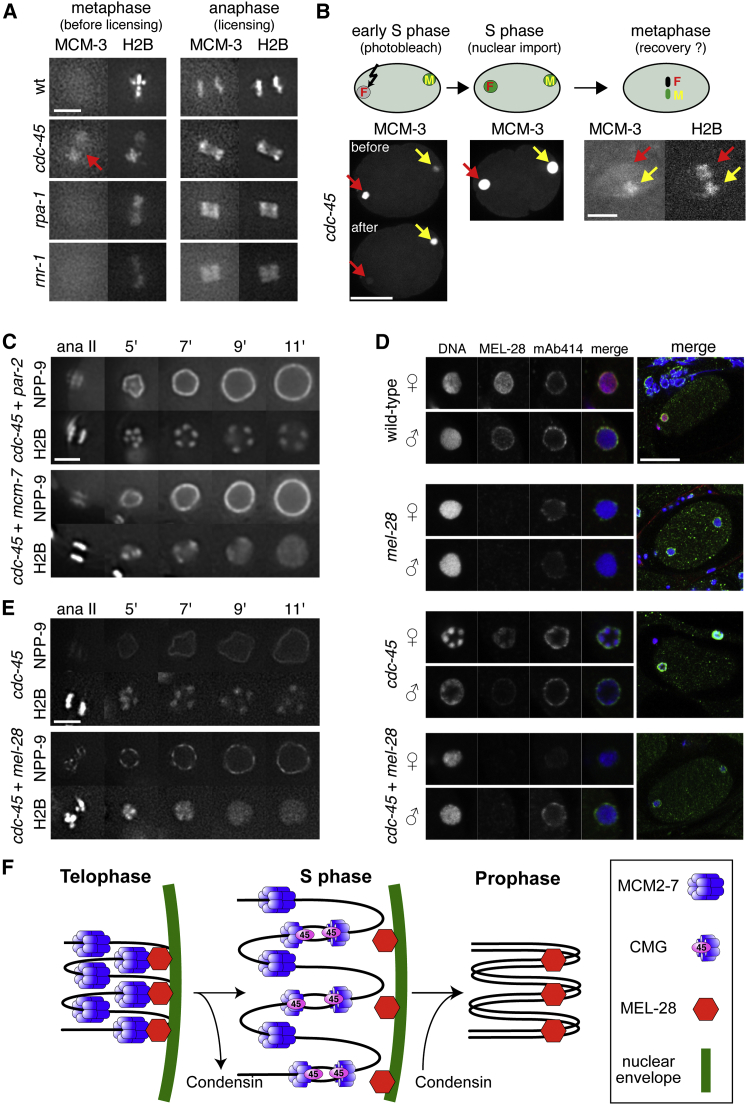
Inactive MCM-2–7 and MEL-28 Prevent the Rapid Chromatin Decondensation (A) GFP-MCM-3 binding to chromatin before and during the licensing period. Images taken from time-lapse sequences of wild-type, *cdc-45*, *rpa-1*, and *rnr-1* RNAi embryos expressing GFP-MCM-3 (left images) and mCherry-H2B (right images) during metaphase (left panel) and anaphase (right panel) of the first embryonic cell cycle. (B) Images from a *cdc-45* RNAi embryo expressing GFP-MCM-3 and mCherry-H2B during the first embryonic cell cycle. The female DNA (red arrows) was photobleached during early S phase (left panel). GFP-MCM-3 gets imported in nuclei (middle panel). Recovery of GFP-MCM-3 was determined during metaphase (right panel). Male chromatin (yellow arrows) served as “unbleached control.” (C) *mcm-7* inactivation partially rescues the *cdc-45* decondensation phenotype. Images taken from time-lapse sequences upon double inactivation of *cdc-45; mcm-7* and *cdc-45; par-2* in embryos expressing GFP-NPP-9 (upper images) and mCherry-H2B (lower images) during the first embryonic S phase. (D) *mel-28* inactivation rescue the *cdc-45* decondensation phenotype. Images of wild-type, *mel-28*, *cdc-45*, and double *cdc-45; mel-28* RNAi embryos during early S phase. Embryos were stained for MEL-28 (red), nuclear pore (mAb414, green), and DNA (blue). Magnifications of the female and male nuclei are shown on the left. (E) Images from *cdc-45* and double *cdc-45; mel-28* RNAi embryos expressing GFP-NPP-9 (top rows) and mCherry-H2B (bottom rows) during the first embryonic S phase. (F) Model for the effect of DNA replication on chromatin structure (see Discussion). Scale bars represent 5 μm (A and B, right panel, and C and E) and 20 μm (B, left panel, and D). Time is expressed in minutes and seconds relative to anaphase II onset. See Movies S7, S8, S9, and S10 for embryos shown in (A)–(C) and (E).

Double-hexameric rings of MCM-2–7 are loaded around origin DNA during the licensing reaction at the end of M phase. These inactive MCM-2–7 complexes disappear from chromatin during the course of S phase, due either to their conversion into active CMG helicase complexes during replication initiation (Blow and Dutta, 2005; Ilves et al., 2010; Moyer et al., 2006) or to their displacement by an active fork. Therefore, when CDC-45 is inactivated, MCM-2–7 double hexamers should remain on DNA throughout the cell cycle, potentially being involved in a condensin-independent mechanism that limits decondensation in *cdc-45* RNAi embryos. Following the initial loading of MCM-2–7 during anaphase (Figure 4H), additional MCM-2–7 complexes are massively imported into nuclei, which obscures the chromatin-bound MCM-2–7 until the following mitosis. In wild-type embryos, chromatin devoid of GFP-MCM-3 can be observed for 1 to 2 min during metaphase at the end of the first cell cycle, from the time of nuclear envelope breakdown until licensing occurs again at the onset of anaphase (Sonneville et al., 2012) (Figure 7A; Movie S7). In contrast, we found that GFP-MCM-3 was still bound to chromatin in *cdc-45* RNAi embryos during early mitosis (Figure 7A; Movie S7), consistent with the idea that when DNA replication was blocked by depletion of CDC-45, inactive MCM-2–7 complexes remained stably on chromatin throughout the cell cycle.

To confirm that the MCM-2–7 on metaphase chromatin in *cdc-45* RNAi was due to persistence of MCM-2–7 complexes on chromatin throughout interphase and was not due to premature loading of MCM-2–7 in early mitosis, we photobleached GFP-MCM-3 during early S phase in female nuclei and determined the recovery in the following mitosis (Figure 7B, red arrows). Male DNA served as a positive control (Figure 7B, yellow arrows). As nuclei increased in size, unbleached GFP-MCM-3 was imported into the photobleached female nucleus (Figure 7B, S phase). After nuclear envelope breakdown at the end of the cell cycle, a GFP-MCM-3 signal was not recovered on bleached chromatin, indicating that GFP-MCM-3 is not loaded prematurely onto metaphase chromatin in *cdc-45* RNAi embryos (Figure 7A) but instead had persisted on chromatin throughout interphase in the absence of replication (Figure 7B; Movie S8).

These experiments raised the possibility that when the initiation of replication has been blocked by *cdc-45* RNAi, the persistence of inactive MCM-2–7 double-hexamers around DNA might support a condensin-independent mechanism that inhibits chromatin decondensation. We therefore simultaneously inactivated *cdc-45* and *mcm-7* to test this hypothesis. As a control for the double RNAi knockdown, we combined *cdc-45* and *par-2* RNAi, the latter gene being required for asymmetric cell division. As anticipated, *cdc-45 par-2* double RNAi resulted both in the severe decondensation defects typical of *cdc-45* RNAi and in the symmetric cell divisions expected for *par-2* RNAi (Figure 7C; Movie S9), confirming the efficiency of the double RNAi approach. We then performed *cdc-45* and *mcm-7* double RNAi and observed the milder decondensation defect that is typical of *mcm-7* RNAi, rather than the severe defect that is normally associated with *cdc-45* inactivation (Figure 7C: Movie S9). This result is consistent with the idea that the severe decondensation defects associated with *cdc-45* RNAi are produced not only by persistence of condensin on chromatin but also by the presence of inactive MCM-2–7 complexes that are loaded tightly around the DNA.

Our results suggest that there are proteins involved in maintaining the condensed state of metaphase chromosomes at the nuclear periphery that are removed, inactivated, or relocalized as a consequence of replication initiation. One possible candidate is ELYS/MEL-28, a nucleoporin that mediates the initial assembly of nuclear pores onto chromosomal DNA (Franz et al., 2007; Gillespie et al., 2007; Rasala et al., 2006) and is also localized to mitotic chromosomes, where it has a function in organizing kinetochores (Fernandez and Piano, 2006; Galy et al., 2006). Importantly, *Xenopus* MEL-28 has been shown to associate with chromatin-bound MCM-2–7 prior to replication initiation (Davuluri et al., 2008; Gillespie et al., 2007). We therefore investigated the idea that removal of MEL-28 from chromatin might be dependent on CDC-45 and the initiation of replication. Consistent with previous reports (Fernandez and Piano, 2006; Franz et al., 2007; Galy et al., 2006), MEL-28 was localized to the nuclear envelope and the nucleoplasm during S phase (Figure 7D). In contrast, when cells were depleted of CDC-45, MEL-28 remained associated with the condensed chromatin (Figure 7D). This is in line with the idea that activation of the CMG helicase is required to release MEL-28 from chromatin as replication initiates.

RNAi against *mel-28* still allowed chromosome decondensation, assembly of the nuclear envelope, and recruitment of the nucleoporins recognized by the mAb414 antibody. To determine whether loss of MEL-28 rescued the chromosome-condensation defect seen upon CDC-45 depletion, cells were treated with RNAi against both *mel-28* and *cdc-45*. Embryo staining (Figure 7D) and live imaging (Figure 7E; Movie S10) showed that co-depletion of MEL-28 and CDC-45 restored the decondensation of the chromatin. Thus, our results suggest that MEL-28 chromatin relocalization contributes to chromosome decondensation and is driven by the initiation of chromosome replication. Consistent with MEL-28 relocalization being stimulated by the loss of MCM-2–7 from chromatin, we found that MEL-28 redistribution occurred similarly after *mcm-7* single and *cdc-45 mcm-7* double RNAi (Figure S3). Our combined data are consistent with the idea that MCM-2–7 complexes dissociate from MEL-28 when MCM-2–7 is either activated or displaced from chromatin, as a consequence of the initiation of DNA replication.

## Discussion

### Dependency of Chromosome Condensation on Prior DNA Replication

We show that if any aspect of DNA replication is inhibited, whether by depleting licensed origins or initiation factors or by inhibiting replication fork elongation, chromosome condensation is defective. Therefore, the condensation defects we observe are not a consequence of the persistence of active replication forks or MCM-2–7 hexamers on the DNA, because condensation defects occur even if MCM-2–7 chromatin loading is inhibited. This phenotype resembles condensin II depletion in *C. elegans* embryos (Csankovszki et al., 2009; Hagstrom et al., 2002; Kaitna et al., 2002; this article), and prompted us to examine condensin localization. The diffuse nuclear localization of the condensin II subunits KLE-2 and HCP-6 prevented us from determining if condensin is loaded onto prophase chromatin when replication is blocked. However, when cells replicate normally, condensin II progressively focuses on chromosomal axes during prophase, and this focusing fails to happen if replication is blocked. Therefore, our results indicate that DNA replication may modify the chromatin such that condensin II promotes chromosome condensation during prophase. This model would be in line with previous reports. In HeLa cells, condensin II gradually accumulates on chromatin during S phase and promotes the separation of replicated DNA (Ono et al., 2013). In *Xenopus* egg extracts, topoisomerase II, which is required for chromosome condensation, associates more tightly with replicated DNA (Cuvier and Hirano, 2003).

Although replication is required for chromosome condensation during prophase, unreplicated chromatin partially condenses during mitosis, leading to an abnormal metaphase plate (Figure 2B; Movie S3). Such a “metaphase condensation” process was previously observed after condensin depletion (Kaitna et al., 2002) and therefore requires neither condensin nor replication.

The replication and condensation defects we observe lead to excessive chromatin bridge formation and a cut phenotype when chromatin is separated during cytokinesis. Such cut phenotypes have been described in fission yeast when mutations affecting replication licensing were first described (Hirano et al., 1986; Hofmann and Beach, 1994; Kelly et al., 1993; Miyake et al., 1993). However, to the best of our knowledge, defects in chromosome condensation have not been described in fission yeast, possibly because the level of DNA condensation could not be assessed easily by DAPI staining of the small chromosomes. In budding yeast, the deletion of genes required for replication initiation leads to a “reductional cell division” occurring with wild-type kinetics and producing two distinct but linked masses of unreplicated DNA in the daughter cells (Piatti et al., 1995; Tercero et al., 2000; Toyn et al., 1995). These phenotypes can be explained by the almost complete absence of replication forks in these mutants, which means that no S phase checkpoint can be generated and, as a consequence, cells enter mitosis on schedule but with unreplicated DNA. Our graded depletion of MCM-7 provides strong support for this interpretation: high levels of depletion led to a cut phenotype with little or no cell-cycle delay, but at lower levels of depletion, presumably associated with partial genome duplication where replication forks are present and capable of activating cell-cycle checkpoints, a pronounced delay of entry into mitosis was observed. However, when these cells ultimately enter mitosis with unreplicated DNA, they nevertheless display a cut phenotype associated with partially condensed DNA.

### Dependency of Chromosome Decondensation on the Initiation of DNA Replication

The rapid cell cycles of embryonic cells have a short or non-existent G1 phase, which means that the initiation of DNA replication happens at about the same time or shortly after decondensation of mitotic chromosomes. In *Xenopus* egg extracts, the initiation of DNA replication occurs only after chromosomes have been incorporated into a functional interphase nucleus (Blow and Sleeman, 1990; Blow and Watson, 1987; Newport, 1987). Chromosome decondensation is largely unaffected when the initiation of DNA replication is inhibited in these extracts (Blow, 1993; Gillespie et al., 2007; Strausfeld et al., 1994). We were therefore surprised to find that in the *C. elegans* embryo, replication commences early, before full chromosome decondensation. However, in vivo studies of the early *Xenopus* embryo also show a similar effect, with DNA replication initiating on only partially decondensed chromosomes (Leibovici et al., 1992; Lemaitre et al., 1998). It appears surprising that DNA replication can occur when chromosomes are in a compacted state. However, DNA synthesis can occur, though at a reduced rate, when DNA is induced to take up a “metaphase-like” condensed form (Gotoh, 2007 and references therein).

We postulate that focusing replication initiation on highly condensed chromatin might aid rapid and synchronous replication. Linking CMG complexes to condensed chromatin and MEL-28 could focus replication closer to the nuclear pore, where imported replication proteins are most accessible (Figure 7F). At the same time, mindful that only a minority of licensed MCM-2–7 double hexamers are activated in any one S phase, initiation on condensed chromatin could also be part of a mechanism facilitating the equal distribution of activated CMG helicases. For example, among the licensed MCM-2–7 located within a condensation loop, the MCM-2–7 complex located closest to the nuclear pore could be preferably activated (Figure 7F). Such a mechanism could ensure that helicase activation is equally distributed along the DNA, thus ensuring the rapid replication of the entire genome.

Our findings also indicate that replication initiation, but not elongation, promotes the rapid decondensation of chromatin. We postulate that the active replicative helicase may induce the release of chromatin-bound complexes, which otherwise maintain condensation. Consistent with this idea, condensin inactivation indeed completely rescued that decondensation defects of MCM-7 depletion. Only a partial rescue occurred when CDC-45 and condensin were co-depleted, in line with our findings that inactive MCM-2–7 and MEL-28 mediate another mode of chromatin compaction in these embryos (as discussed below). The requirement of CMG activation, but not replication fork progression, for decondensation can be explained in two different ways. The first possibility is that factors regulating condensation colocalize with MCM-2–7 on chromatin, as has been shown in *Xenopu*s for MEL-28 (Gillespie et al., 2007), so that conversion of MCM-2–7 to the CMG could allow decondensation. A second explanation is that DNA unwinding by the CMG helicase could displace condensins from DNA and allow decondensation.

It has been previously shown in *Xenopus* that ELYS/MEL-28 interacts on chromatin with MCM-2–7 (Davuluri et al., 2008; Gillespie et al., 2007). In the *Xenopus* early embryo, ELYS/MEL-28 dependent nuclear assembly shuts down the replication licensing system (Hodgson et al., 2002; Kisielewska and Blow, 2012), to create a feed-forward loop that accelerates nuclear assembly and S phase entry once origin licensing is complete. There is also evidence in the *Xenopus* system that DNA replication promotes decondensation of mitotically condensed chromatin (Prokhorova et al., 2003). If a similar MCM-MEL-28 interaction also occurs in the *C. elegans* embryo (Figure 7B, telophase), this would explain why depletion of MCM-7 rescues the decondensation defect that occurs in the absence of CDC-45. Under this interpretation, the initiation of DNA replication, which is dependent on both nuclear assembly and CDC-45, would promote dissociation of MCMs from MEL-28, thereby allowing the DNA to rapidly decondense once replication has initiated (Figure 7F, S phase). Consistent with this interpretation, we find that MEL-28 remained associated with condensed chromatin when CDC-45 was depleted. The possible CDC-45-dependent extraction of MEL-28 from chromatin would therefore be part of a mechanism that links replication licensing to nuclear assembly and chromosome decondensation.

With the sole exception of the spindle assembly checkpoint, the cell-cycle engine of somatic cells, powered by CDK activity, is not strongly influenced by feedback from cell-cycle events occurring normally (i.e., in the absence of contingencies such as DNA damage). We show here that in the short cell cycles of *C. elegans* embryonic cells, chromosome decondensation, replication licensing, DNA replication, and chromosome recondensation all occur in a tightly coordinated manner. This coupling helps ensure the correct order of cell-cycle events and the faithful propagation of chromosomal DNA, which is particularly important for embryonic cells as they ultimately contribute to the germline and DNA of future generations.

## Experimental Procedures

### *C. elegans* Maintenance and RNAi

*C. elegans* were maintained according to standard procedures (Brenner, 1974). RNAi was performed by feeding worms with bacteria expressing double-stranded RNA (Timmons and Fire, 1998). Details about worms and RNAi can be found in Supplemental Experimental Procedures.

### Microscopy

Different procedures were used to record embryos from the first embryonic cell cycle or from meiotic divisions, and embryos were imaged using confocal or widefield fluorescent microscopy (see Supplemental Experimental Procedures). For immunostaining, embryos were fixed in methanol at −20°C and stained using standard procedures with rabbit antibodies for phospho-H3S10 (1:1,200; Upstate), MEL-28 (1:500) (Galy et al., 2006), and HCP-6 (1:500) (Hargitai et al., 2009) and with mouse monoclonal antibodies mAb414 (1:200; Covance). Secondary antibodies were donkey anti-rabbit conjugated to Alexa Fluor 568 (Invitrogen) and donkey anti-mouse conjugated with Alexa 488 (Invitrogen). DNA was visualized with Hoechst 33258. Embryos were imaged using a confocal laser-scanning microscope (SP2; Leica) using a 63×/1.40 plan-apochromat oil-immersion lens (Leica), except for EdU staining, in which a DeltaVision Core microscope was used (see details above).

### EdU Incorporation

In Figure 1, embryos at meiosis II, permeabilized by *perm-1* (diluted 1/6) RNAi (Carvalho et al., 2011), were incubated for 25–35 min in isotonic buffer (see Supplemental Experimental Procedures) + 20 μM EdU and incubated for 25–35 min. Embryos at first embryonic mitosis were fixed in 3.6% formaldehyde; permeabilized in PBS, 0.5% Triton X-100 for 20 min; and washed in PBS. EdU incorporation was revealed with Click-iT EdU Alexa 488 Imaging Kit (Invitrogen). DNA was stained with Hoechst 33342, and embryos were mounted on polylysine-coated slides. In Figures 2 and 4, embryos were dissected from ten worms and incubated in isotonic buffer supplemented with 20 μM EdU for 30 min (Figure 2), 10 min (Figure 4A), or 5 min (Figure 4D) and then treated as described above.

### Image Quantification

ImageJ was used for all quantifications. GFP enrichment on mitotic DNA was determined using middle plan images during metaphase and calculated by subtracting the integrated density of an area containing cytoplasm from the integrated density of a similar area containing the chromatin. Values were normalized to wild-type levels. The same procedure was applied for the quantification of EdU staining, except that the image was the intensity sum of 15 z stacks acquired every 0.5 μm.

## Author Contributions

R.S. was responsible for designing and conducting all the experiments and supervising G.C. R.S., A.G., K.L., and J.J.B. planned the research strategy, analyzed the data, and wrote the manuscript.

## Supplemental Information

Document S1. Supplemental Experimental Procedures, Figures S1–S3, and Table S1

Movie S1. Video of an Embryo Expressing GFP-NPP-9 and mCherry-Histone Progressing throughout the Second Meiotic Division and First Embryonic Cell Cycle, Related to Figure 1A

Movie S2. Composite Video Showing Wild-Type and *mcm-7* RNAi Embryos Expressing GFP-NPP-9 and mCherry-Histone Progressing throughout the Second Meiotic Division and First Embryonic Cell Cycle, Related to Figure 1B

Movie S3. Composite Video Showing Wild-Type, *cdt-1* RNAi, *mcm-7* RNAi, *cdc-45* RNAi, *rpa-1* RNAi, *rnr-1* RNAi, and *smc-4* RNAi Embryos Expressing GFP-Histone Progressing throughout the First Embryonic Cell Cycle, Related to Figure 2B

Movie S4. Composite Video Showing, from Top to Bottom, Wild-Type, *mcm-7*, *cdc-45*, and *rpa-1* RNAi Embryos Expressing GFP-KLE-2 and mCherry-Histone Progressing throughout Prophase of the First Embryonic Cell Cycle, Related to Figure 2D

Movie S5. Composite Video Showing, from Top to Bottom, Wild-Type, *mcm-7* RNAi, *cdc-45* RNAi, and *rpa-1* RNAi Embryos Expressing GFP-NPP-9 and mCherry-Histone Progressing throughout the Second Meiotic Division and First Embryonic S Phase, Related to Figure 5A

Movie S6. Composite Video Showing, from Top to Bottom, *cdc-45*, Double *cdc-45*; *smc-4*, *mcm-7*, Double *mcm-7*; *smc-4*, and *smc-4* RNAi Embryos Expressing GFP-CAPG-1 and mCherry-Histone Progressing throughout the Second Meiotic Division and First Embryonic Cell Cycle, Related to Figure 6B

Movie S7. Composite of Videos Showing, from Top to Bottom, Wild-Type, *cdc-45* RNAi, *rpa-1* RNAi, and *rnr-1* RNAi Embryos Expressing GFP-MCM-3 and mCherry-Histone Progressing throughout the First Embryonic Mitosis, Related to Figure 7A

Movie S8. Video of an Embryo Expressing GFP-MCM-3 and mCherry-Histone Progressing throughout the First Embryonic Cell Cycle, Related to Figure 7B The female nuclei were photobleached during early S phase.

Movie S9. Composite Video Showing Embryos Doubly Inactivated for *cdc-45*/*par-2* and *cdc-45*/*mcm-7*, Related to Figure 7C Embryos are expressing GFP-NPP-9 and mCherry-Histone progressing throughout the second meiotic division and first embryonic cell cycle.

Movie S10. Composite Video Showing *cdc-45* and Double *cdc-45*; *mel-28* RNAi Embryos Expressing GFP-NPP-9 and mCherry-Histone Progressing throughout the Second Meiotic Division and First Embryonic Cell Cycle, Related to Figure 7E

Document S2. Article plus Supplemental Information
