# Supplementary material for: Both Chromosome Decondensation and Condensation Are Dependent on DNA Replication in C. elegans Embryos
Source: Cell Rep. 2015 Jul 9;12(3):405–17. doi: 10.1016/j.celrep.2015.06.046 (PMC4521082; doi:10.1016/j.celrep.2015.06.046)
Supplement: Document S1. Supplemental Experimental Procedures, Figures S1–S3, and Table S1 [file mmc1.pdf]

Cell Reports
Supplemental Information

# Both Chromosome Decondensation and Condensation Are Dependent on DNA Replication in *C. elegans* Embryos

Remi Sonneville, Gillian Craig, Karim Labib, Anton Gartner, and J. Julian Blow

Supplemental information

Supplemental Experimental Procedures

Strains

The following worms strains were used: Bristol N2, TG3827 *unc-119(ed3) III; axIs1595[pie-1p::gfp::npp-9 + unc-119(+)]; ltIs37[pie-1p::mCherry::his-58+ unc-119(+)]* (Voronina and Seydoux, 2010), TG3825 *unc-119(ed3) III; ojIs50[pie-1p::gfp::air-2 + unc-119(+)]; ltIs37* (de Carvalho et al., 2008), EKM36 *unc-119(ed3) III; cldIs[pie-1p::gfp::capg-1 + unc-119(+)]; ltIs37* (Collette et al., 2011), TG2512 *unc-119(ed3) III; gtIs2512[pie-1p::gfp::his-11 + unc-119(+)],* TG1753 *unc-119(ed3) III; gtIs64[pie-1p::gfp::mcm-3 + unc-119(+)]; ltIs37*, TG1754 *unc-119(ed3) III; gtIs65[pie-1p::gfp::cdc-45 + unc-119(+)]; ltIs37*, TG2368 *unc-119(ed3) III; gtIs2368[pie-1p::gfp::rpa-1 + unc-119(+)]; ltIs37*, TG3828 *unc-119(ed3) III; gtIs3828[pie-1p::gfp::kle-2 + unc-119(+)]; ltIs37* (Sonneville et al., 2012). Transgenic worms expressing GFP-tagged KLE-2 were generated by particle bombardment (Praitis et al., 2001); the full-length cDNA was amplified from N2 worms and cloned into *pie-1* regulatory element in a pIC26 vector (Cheeseman and Desai, 2005).

RNAi

Clones for *mcm-7*, *rpa-1*, *rnr-1*, *perm-1*, *npp-8*, *mel-28* and *par-2* feeding strains were obtained from a commercial library (Kamath et al., 2003). Clones for *cdt-1*, *cdc-45*, *smc-4* and *hcp-6* feeding strains were generated by cloning cDNAs into L4440. Bacteria transformed with an empty L4440 vector were used as control throughout this study. The following oligonucleotides were used for amplification: *cdt-1* 5' atgagttcccgggtgactag, *cdt-1* 3' ttaatgaaatttgagagatcttgc, *cdc-45* 5' atgatcatcgaagacaactttcgg, *cdc-45* 3' tcagttggatgtgccctca, *smc-4* 5' atgcctccgaagacttcggc, *smc-4* 3' ttattccatatcatcatcgtcatc, *hcp-6* 5' cactagtagccggagtaaaatccttgt, *hcp-6* 3' cactagtctggcatgttcagtgacgtc. To make RNAi plates, bacteria were grown to OD_{600} = 1, supplemented with 1 mM IPTG, and spread on 10 ml RNAi-plates (3 g/l NaCl, 2% agarose, 1 mM $CaCl_2$, 1 mM $MgSO_4$, 25 mM potassium phosphate pH 6.5 mg/l cholesterol). dsRNA was induced 16 hours at room temperature. L4 worms were then added to RNAi-plates and fed for 26–32 hours at 25°C before analysis. For double RNAi, equal amounts of bacterial cultures were mixed together prior to seeding the RNAi plates.

Microscopy

To record meiotic divisions, embryos were dissected in a previously described isotonic growth medium for blastomeres containing 35% bovine FCS (Shelton and Bowerman, 1996): 1 ml of 5 mg/ml inulin, 50 mg tissue culture–grade polyvinylpyrrolidone (Sigma-Aldrich), 100 µl Basal Medium Eagle vitamins (Invitrogen), 100 µl chemically defined lipid concentrate (Invitrogen) and 100× concentrated penicillin-streptomycin (Invitrogen) and 9 ml *Drosophila* Schneider's Medium (Invitrogen). Before use, bovine FCS (Invitrogen) (heat treated for 30 min at 56°C) was added. Embryos were mounted on 2% agarose pads, and Vaseline patches on the slide reduced the pressure of the coverslip on the embryo. Embryos, recorded from the first embryonic cell cycle, were dissected in M9 (3 g/l KH_2PO_4, 6 g/l Na_2HPO_4, 5 g/l NaCl, 1 mM $MgSO_4$) and mounted on 2% agarose pads. Images were captured every 10 s or 30 s using a widefield DeltaVision Core microscope mounted on a microscope (IX71; Olympus) with a 60×/1.40 Plan Apochromat oil immersion lens (Olympus), a camera (CoolSNAP HQ; Photometrics), and softWoRx software. The exposure time was 0.25 s. Images were deconvolved using softWoRx software. Embryos expressing GFP–CDC-45, GFP-CAPG-1 and GFP-MCM-3 (in photobleaching experiments) were recorded using a spinning-disk confocal microscope (MAG Biosystems) mounted on a microscope (IX81; Olympus) with a 60×/1.40 Plan Apochromat oil immersion lens (Olympus), a camera (Cascade II;

Photometrics), spinning-disk head (CSU-X1; Yokogawa Electric Corporation), and MetaMorph software (Molecular Devices). Photobleaching of female nuclei were done with a 200ms pulse 488nm laser using iLas2 system (Roger Scientific). The light path was modified after photobleaching to optimized image acquisition but resulting in a slight shift of the image position. Embryos were recorded at 23–24°C. Image analysis and video processing were performed using ImageJ software (National Institutes of Health). For immunostaining, embryos were fixed in methanol at −20°C and stained using standard procedures with rabbit antibodies for phospho-H3S10 (1:1,200; Upstate), MEL-28 (1:500) (Galy et al., 2006), HCP-6 (1:500) (Hargitai et al., 2009) and with mouse monoclonal antibodies mAb414 (1:200; Covance). Secondary antibodies were donkey anti–rabbit conjugated to Alexa Fluor 568 (Invitrogen) and donkey anti–mouse conjugated with Alexa Fluor 488 (Invitrogen). DNA was visualized with Hoechst 33258. Embryos were imaged using a confocal laser-scanning microscope (SP2; Leica) using a 63×/1.40 Plan Apochromat oil immersion lens (Leica), except for EdU staining, in which a DeltaVision Core microscope was used (see details above).

Supplemental Figures

Figure S1. Localization of AIR-2 and KLE-2 during the first embryonic cell cycle, related to Figure 2.

(A) Nuclear GFP-AIR-2 during prophase. Images taken from time-lapse sequences of wild-type, *mcm-7*, *cdc-45*, *rnr-1* and *smc-4* RNAi embryos expressing GFP-AIR-2 (upper images) and mCherry-Histone H2B (lower images) during the first embryonic cell cycle. (B) Quantification of GFP-AIR-2 on chromatin during metaphase of mitosis. Average values of 5 embryos prepared as in A. (C) Time duration between meeting of nuclei and prophase entry as determined by GFP-AIR-2 nuclear entry from 5 embryos. (D) GFP-KLE-2 and condensin II. Images from wild-type, *smc-4* and *hcp-6* RNAi embryos expressing GFP-KLE-2 (top images) and mCherry-Histone H2B (bottom images) during prophase and metaphase. Scale bars are 5 µm. Error bars represent SD.

Figure S2. Assembly of a functional nuclear envelope upon DNA replication block, related to Figure 4.

(A) Localization of GFP-RPA-1. Images taken from time-lapse sequences of wild-type and *cdc-45* RNAi embryos expressing GFP-RPA-1 (top images) and mCherry-Histone H2B (middle images), and merged GFP-RPA-1 (green) and mCherry-Histone H2B (red) (bottom images) during early S phase. (B) Cartoon representation of nuclei shown in A at 5'30 after anaphase II onset. (C) Duration of the time period from anaphase II onset to the assembly of GFP-NPP-9 ring around the chromatin, as summarized in the scheme underneath, in wild-type, *cdc-45*, *mcm-7*, *cdt-1* and *rpa-1* RNAi embryos. Bars represent the mean values for 5 embryos prepared as in Figure 5A. (D) Duration of the time period from anaphase II onset to nuclear GFP-RPA-1 or GFP-MCM-3, in wild-type and *cdc-45* RNAi embryos, as summarized in the scheme underneath. Bars represent the mean values for 5 embryos prepared as in A (GFP-RPA-1) or as in Figure 4C (GFP-MCM-3). Scale bar is 5 µm. Error bars represent SD.

Figure S3. inactive MCM2-7 and MEL-28 maintain chromatin condensation, related to Figure 7.

Visualization of MEL-28 during early S phase. *cdc-45*, double *cdc-45*; *mcm-7* and *mcm-7* RNAi embryos were stained for MEL-28 (red), nuclear pores (mAb414, green) and DNA (blue). Nuclei imaged with a higher magnification are shown on the left.

Supplemental Movies

Movie S1 - related to Figure 1A. Video of an embryo expressing GFP–NPP-9 (left) and mCherry-Histone (right) progressing throughout the second meiotic division and the first embryonic cell cycle. Images were acquired every 30 s with a widefield fluorescent microscope (DeltaVision; Applied Precision) and deconvolved with Softworks. Time is indicated in minutes and seconds.

Movie S2 - related to Figure 1B. Composite video showing wild-type (top) and *mcm-7* RNAi (bottom) embryos expressing GFP–NPP-9 (left) and mCherry-Histone (right) progressing throughout the second meiotic division and the first embryonic cell cycle. Videos were synchronized to the onset of anaphase II. Images were acquired every 30 s with a widefield fluorescent microscope (DeltaVision; Applied Precision) and deconvolved with Softworks. Time is indicated in minutes and seconds.

Movie S3 - related to Figure 2B. Composite video showing wild-type, *cdt-1* RNAi, *mcm-7* RNAi, *cdc-45* RNAi, *rpa-1* RNAi, *rnr-1* RNAi and *smc-4* RNAi embryos expressing GFP–Histone progressing throughout the first embryonic cell cycle. Videos were synchronized to the meeting of the two nuclei. Images were acquired every 10 s with a spinning-disk confocal fluorescent microscope (MAG Biosystems). Time is indicated in minutes and seconds.

Movie S4 - related to Figure 2D. Composite video showing, from top to bottom, wild-type, *mcm-7*, *cdc-45* and *rpa-1* RNAi embryos expressing GFP–KLE-2 (left) and mCherry-Histone (right) progressing throughout prophase of the first embryonic cell cycle. Videos were synchronized to nuclei meeting. Images were acquired every 10 s with a spinning-disk confocal fluorescent microscope (MAG Biosystems). Time is indicated in minutes and seconds.

Movie S5 - related to Figure 5A. Composite video showing from top to bottom, wild-type, *mcm-7* RNAi, *cdc-45* RNAi, *rpa-1* RNAi embryos expressing GFP–NPP-9 (left) and mCherry-Histone (right) progressing throughout the second meiotic division and the first embryonic S phase. For simplicity, only the anterior of embryos are shown. Videos were synchronized to the onset of anaphase II. Images were acquired every 30 s with a widefield fluorescent microscope (DeltaVision; Applied Precision) and deconvolved with Softworks. Time is indicated in minutes and seconds.

Movie S6 - related to Figure 6B. Composite video showing, from top to bottom *cdc-45*, double *cdc-45*; *smc-4*, *mcm-7*, double *mcm-7*; *smc-4* and *smc-4* RNAi embryos expressing GFP–CAPG-1 (left) and mCherry-Histone (right) progressing throughout the second meiotic division and the first embryonic cell cycle. Videos were synchronized to the anaphase II onset. Images were acquired every 30 s with a spinning-disk confocal fluorescent microscope (MAG Biosystems). Time is indicated in minutes and seconds.

Movie S7 - related to Figure 7A. Composite of videos showing from top to bottom, wild-type, *cdc-45* RNAi, *rpa-1* RNAi and *rnr-1* RNAi, embryos expressing GFP–MCM-3 (left) and mCherry-Histone (right) progressing throughout the first embryonic mitosis. Videos were synchronized to the time of nuclear envelope breakdown. Images were acquired every 10 s with a widefield fluorescent microscope (DeltaVision; Applied Precision) and deconvolved with Softworks. Time is indicated in minutes and seconds.

Movie S8 - related to Figure 7B. Video of an embryo expressing GFP–MCM-3 (left) and mCherry-Histone (right) progressing throughout the first embryonic cell cycle. The female nuclei was photobleached during early S phase (at 00:50), GFP-MCM-3 chromatin recovery can be determined from 20:10 until 21:40, when commences the next licensing period. Images were acquired every 10 s but only few images were taken during late S phase and prophase to minimized photobleaching. The experiment was performed with a spinning-disk confocal fluorescent microscope (MAG Biosystems) and a laser using iLas2 system (Roger Scientific). Time is indicated in minutes and seconds.

Movie S9 - related to Figure 7C. Composite video showing embryos doubly inactivated for *cdc-45*; *mcm-7* (top) and *cdc-45*; *par-2* (bottom). Embryos are expressing GFP–NPP-9 (left) and mCherry-Histone (right) progressing throughout the second meiotic division and the first embryonic cell cycle. Videos were synchronized to the anaphase II onset. Images were acquired every 30 s with a widefield fluorescent microscope (DeltaVision; Applied Precision) and deconvolved with Softworks. Time is indicated in minutes and seconds.

Movie S10 - related to Figure 7E. Composite video showing *cdc-45* (top) and double *cdc-45*; *mel-28* (bottom) RNAi embryos expressing GFP–NPP-9 (left) and mCherry-Histone (right) progressing throughout the second meiotic division and the first embryonic cell cycle. Videos were synchronized to the anaphase II onset. Images were acquired every 30 s with a widefield fluorescent microscope (DeltaVision; Applied Precision) and deconvolved with Softworks. Time is indicated in minutes and seconds.

Table S1 - related to Figure 2.

	H. sapiens	*C. elegans* homologues (from wormbase.org)		Phenobank	
Function	Replication gene	locus	sequence	segregation defects ('cut phenotype') at 1st mitosis (our observation)	embryonic lethal
pre-RC	*orc1*	*orc-1*	Y39A1A.12	no	no
	orc2	*orc-2*	F59E10.1	no	yes
	orc3	*orc-3*	Y119D3B.11	n.a.	no
	orc4	*orc-4*	Y39A1A.13	no	no
	orc5	*orc-5*	ZC168.3	n.a.	no
	cdc6	*cdc-6*	C43E11.10	YES	yes
	cdt1	*cdt-1*	Y54E10A.15	YES	yes
	mcm2	*mcm-2*	Y17G7B.5	YES	yes
	mcm3	*mcm-3*	C25D7.6	YES	yes
	mcm4	*mcm-4*	Y39G10AR.14	YES	yes
	mcm5	*mcm-5*	R10E4.4	YES	yes
	mcm6	*mcm-6*	ZK632.1	YES	yes
	mcm7	*mcm-7*	F32D1.10	YES	yes
initiation	*cdc7*		C34G6.5	no	no
	ticrr		ZK484.4 *	n.a.	no
	recql4		T12F5.1	n.a.	no
	topbp1	*mus-101*	F37D6.1	no	no
	mcm10	*mcm-10*	Y47D3A.28	no	no
	cdc45	*evl-18*	F34D10.2	YES	yes
	sld5	*sld-5*	Y113G7B.24	no	yes
	psf1	*psf-1*	R53.6	no	yes
	psf2	*psf-2*	F31C3.5	no	yes
	psf3	*psf-3*	Y65B4BR.8	no	yes
	rpa1	*rpa-1*	F18A1.5	YES	yes
	rpa2	*rpa-2*	M04F3.1	no	no
	pcna	*pcn-1*	W03D2.4	YES	yes
	rfc1		C54G10.2	no	yes
	rfc2		F58F6.4	no	yes
	rfc3		C39E9.13	no	yes
	rfc4		F31E3.3	no	yes
	rfc5		F44B9.8	no	yes

	prim1	*pri-1*	F58A4.4	no	yes
	prim2	*pri-2*	W02D9.1	no	yes
elongation	*pola1*		Y47D3A.29	no	yes
	pola2	*div-1*	R01H10.1	no	yes
	rrm1	*rnr-1*	T23G5.1	YES	yes
	rrm2	*rnr-2*	C03C10.3	YES	yes
	pold1		F10C2.4	weak	yes
	pold2		F12F6.7 **	no	yes
	pole2	*pole-2*	F08B4.5	no	yes

Compilation of phenotypes observed upon depletion of conserved DNA replication genes as deposited in phenobank (http://www.worm.mpi-cbg.de/phenobank/cgi-bin/MenuPage.py.). 'emb.' stands for embryonic lethal; n.a for not applicable;* via bioinformatic evidence (Sanchez-Pulido et al., 2010); ** obtained by reciprocal blast.

Reference

Cheeseman, I.M., and Desai, A. (2005). A combined approach for the localization and tandem affinity purification of protein complexes from metazoans. Sci STKE *2005*, pl1.

Collette, K.S., Petty, E.L., Golenberg, N., Bembenek, J.N., and Csankovszki, G. (2011). Different roles for Aurora B in condensin targeting during mitosis and meiosis. Journal of cell science *124*, 3684-3694.

de Carvalho, C.E., Zaaijer, S., Smolikov, S., Gu, Y., Schumacher, J.M., and Colaiacovo, M.P. (2008). LAB-1 antagonizes the Aurora B kinase in C. elegans. Genes & development *22*, 2869-2885.

Galy, V., Askjaer, P., Franz, C., Lopez-Iglesias, C., and Mattaj, I.W. (2006). MEL-28, a novel nuclear-envelope and kinetochore protein essential for zygotic nuclear-envelope assembly in C. elegans. Current biology : CB *16*, 1748-1756.

Hargitai, B., Kutnyanszky, V., Blauwkamp, T.A., Stetak, A., Csankovszki, G., Takacs-Vellai, K., and Vellai, T. (2009). xol-1, the master sex-switch gene in C. elegans, is a transcriptional target of the terminal sex-determining factor TRA-1. Development *136*, 3881-3887.

Kamath, R.S., Fraser, A.G., Dong, Y., Poulin, G., Durbin, R., Gotta, M., Kanapin, A., Le Bot, N., Moreno, S., Sohrmann, M*., et al.* (2003). Systematic functional analysis of the Caenorhabditis elegans genome using RNAi. Nature *421*, 231-237.

Praitis, V., Casey, E., Collar, D., and Austin, J. (2001). Creation of low-copy integrated transgenic lines in Caenorhabditis elegans. Genetics *157*, 1217-1226.

Sanchez-Pulido, L., Diffley, J.F., and Ponting, C.P. (2010). Homology explains the functional similarities of Treslin/Ticrr and Sld3. Current biology : CB *20*, R509-510.

Shelton, C.A., and Bowerman, B. (1996). Time-dependent responses to glp-1-mediated inductions in early C. elegans embryos. Development *122*, 2043-2050.

Sonneville, R., Querenet, M., Craig, A., Gartner, A., and Blow, J.J. (2012). The dynamics of replication licensing in live Caenorhabditis elegans embryos. The Journal of cell biology *196*, 233-246.

Voronina, E., and Seydoux, G. (2010). The C. elegans homolog of nucleoporin Nup98 is required for the integrity and function of germline P granules. Development *137*, 1441-1450.

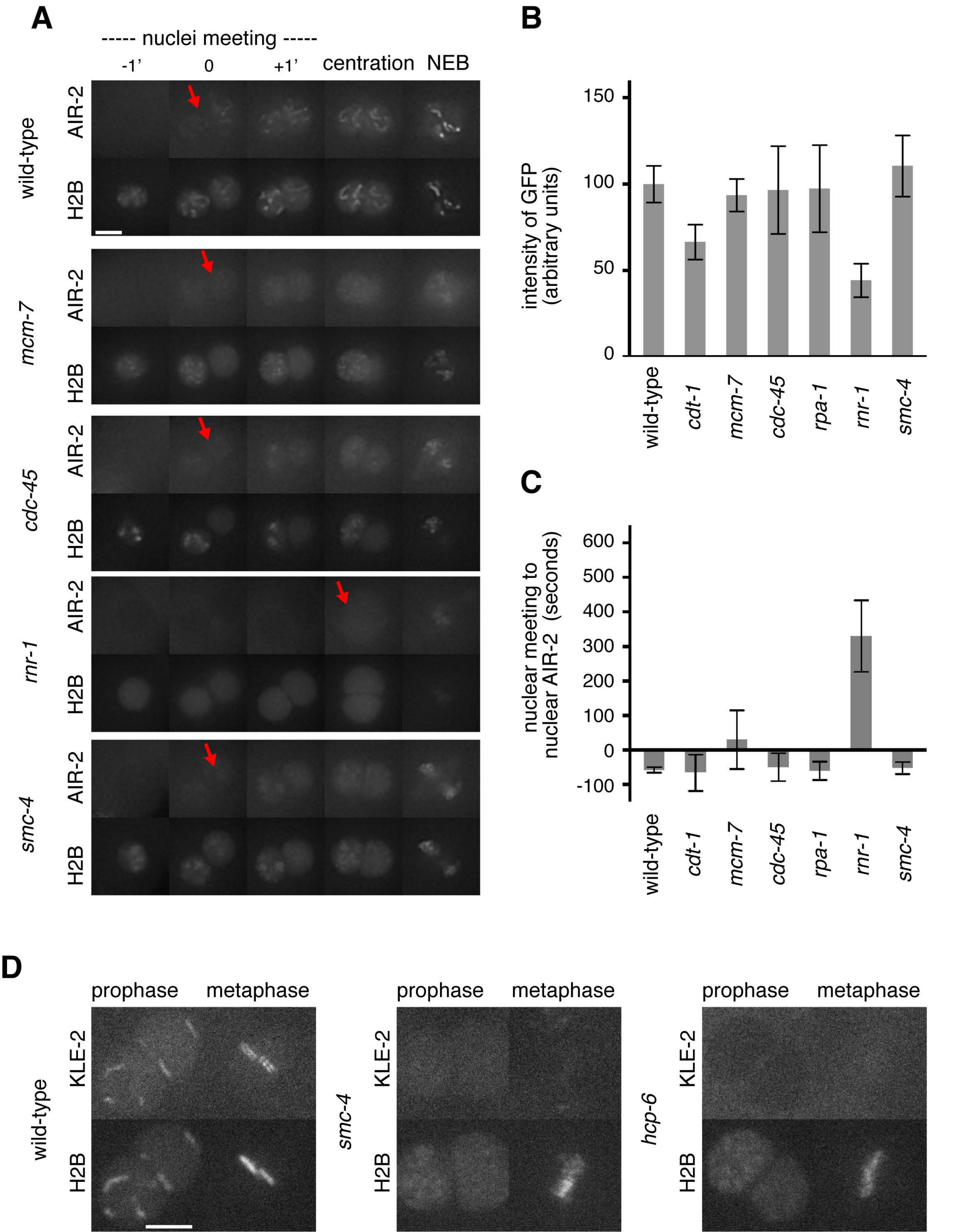
A
----- nuclei meeting -----
-1'
0
+1'
centration
NEB
wild-type
mcm-7
cdc-45
rnr-1
smc-4
AIR-2
H2B
B
intensity of GFP (arbitrary units)
150
100
50
0
wild-type
cdt-1
mcm-7
cdc-45
rpa-1
rnr-1
smc-4
C
nuclear meeting to nuclear AIR-2 (seconds)
600
500
400
300
200
100
0
-100
D
prophase
metaphase
KLE-2
H2B
wild-type
smc-4
hcp-6

Sonneville et al, Supplementary Figure S2

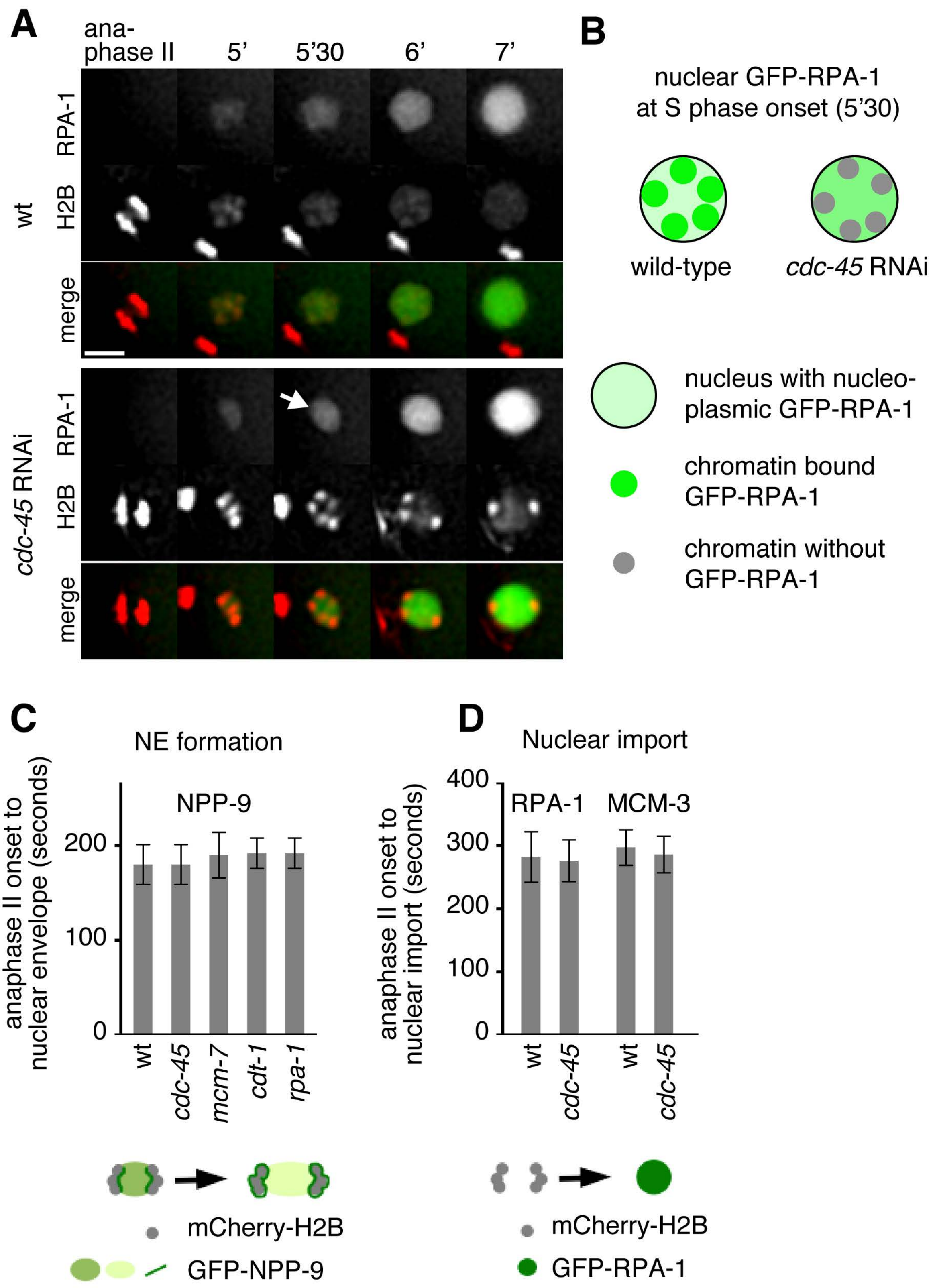

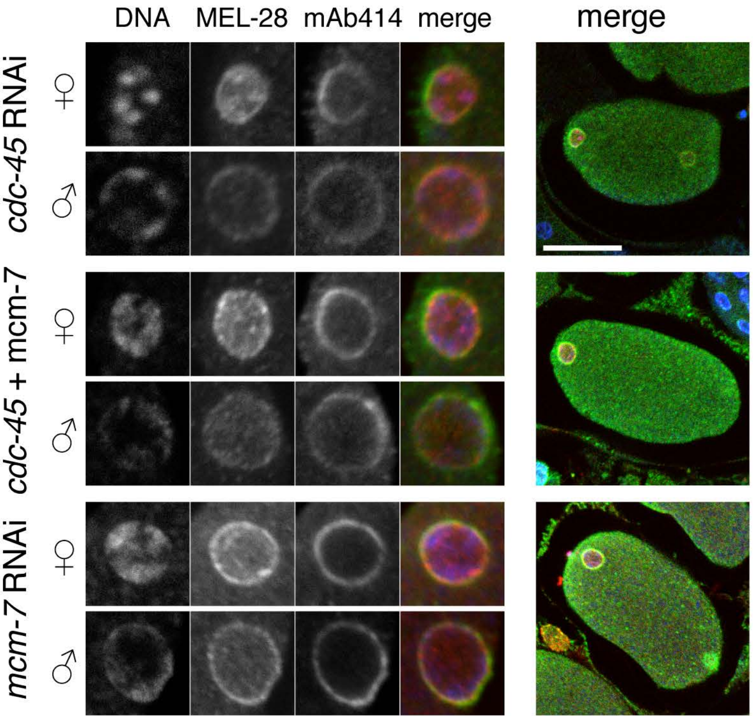
DNA
MEL-28
mAb414
merge
merge
cdc-45 RNAi
cdc-45 + mcm-7
mcm-7 RNAi
♀
♂
