# Supplementary figures and images for: Both Chromosome Decondensation and Condensation Are Dependent on DNA Replication in C. elegans Embryos
Source: Cell Rep. 2015 Jul 9;12(3):405–17. doi: 10.1016/j.celrep.2015.06.046 (PMC4521082; doi:10.1016/j.celrep.2015.06.046)

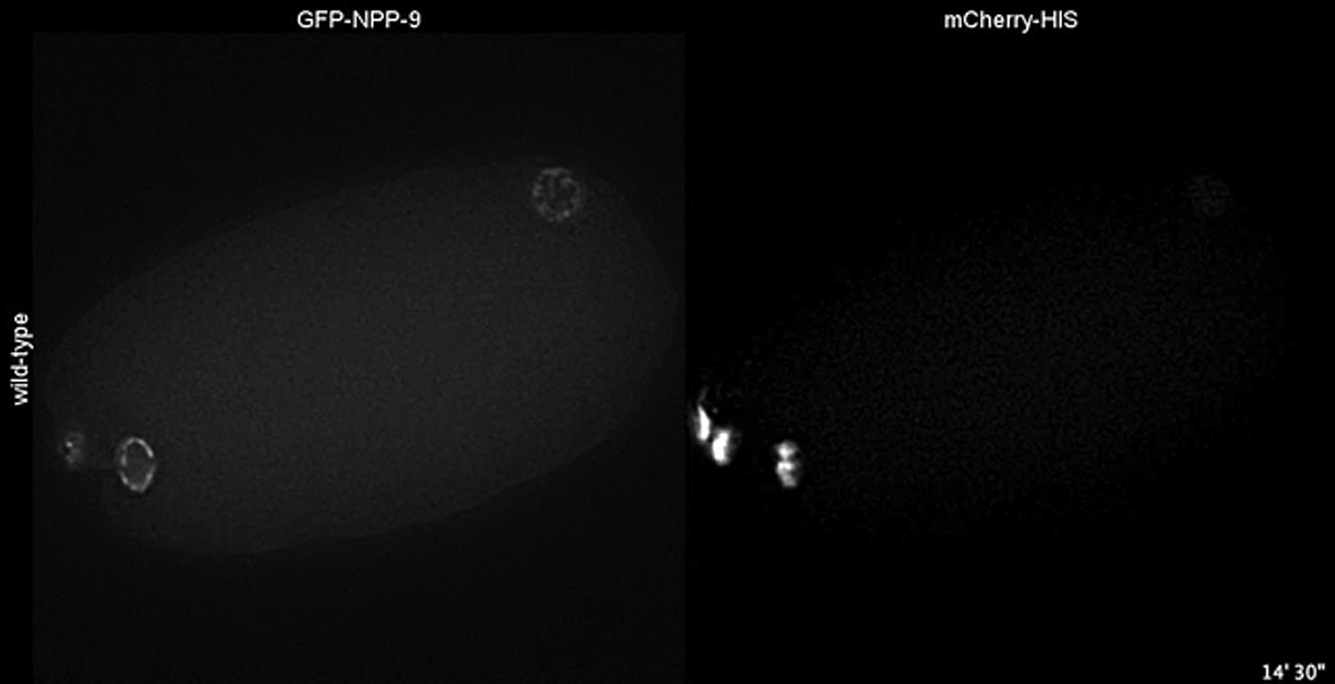

Supplement: Movie S1. Video of an Embryo Expressing GFP-NPP-9 and mCherry-Histone Progressing throughout the Second Meiotic Division and First Embryonic Cell Cycle, Related to Figure 1A [file mmc2.jpg]

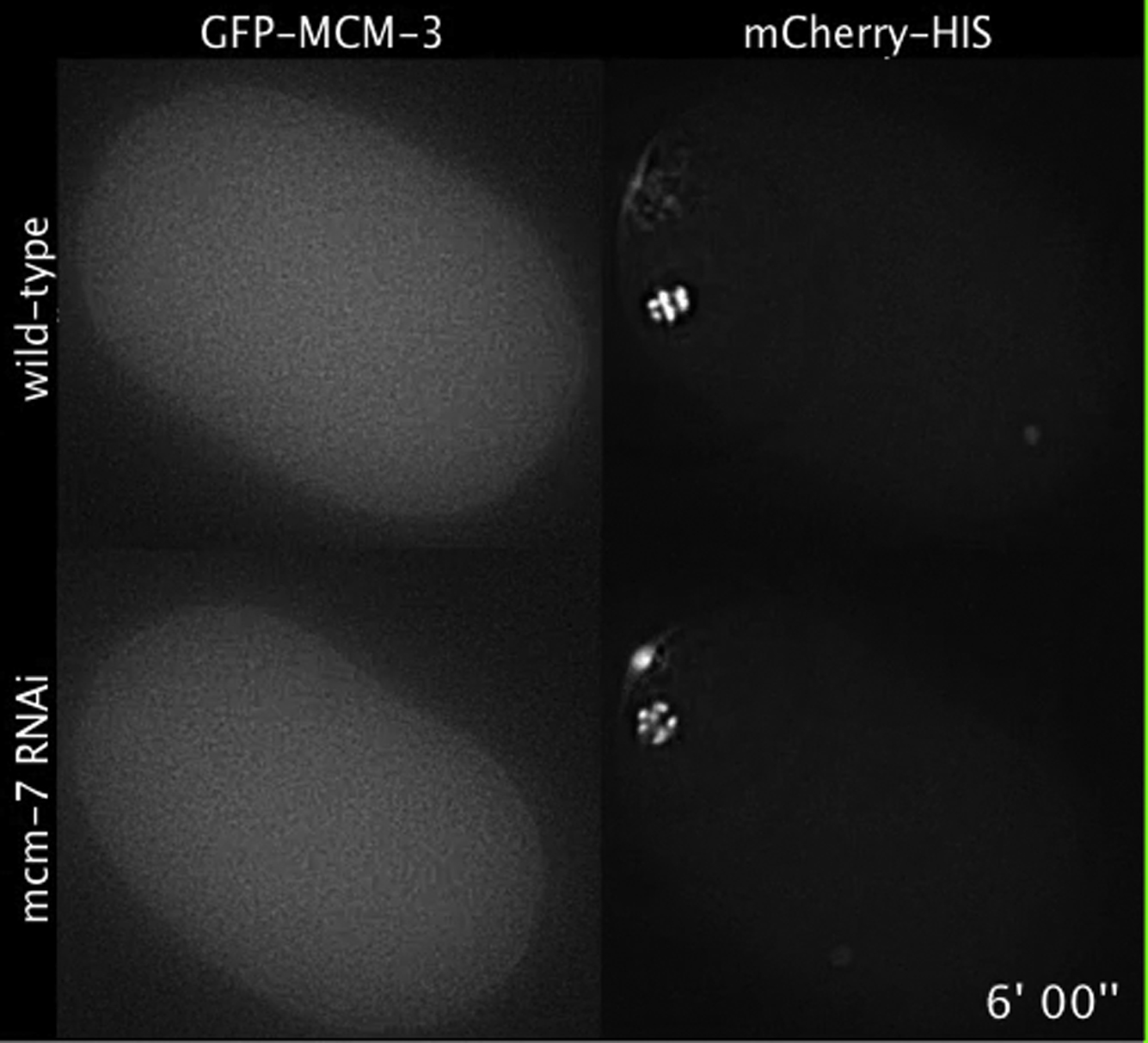

Supplement: Movie S2. Composite Video Showing Wild-Type and mcm-7 RNAi Embryos Expressing GFP-NPP-9 and mCherry-Histone Progressing throughout the Second Meiotic Division and First Embryonic Cell Cycle, Related to Figure 1B [file mmc3.jpg]

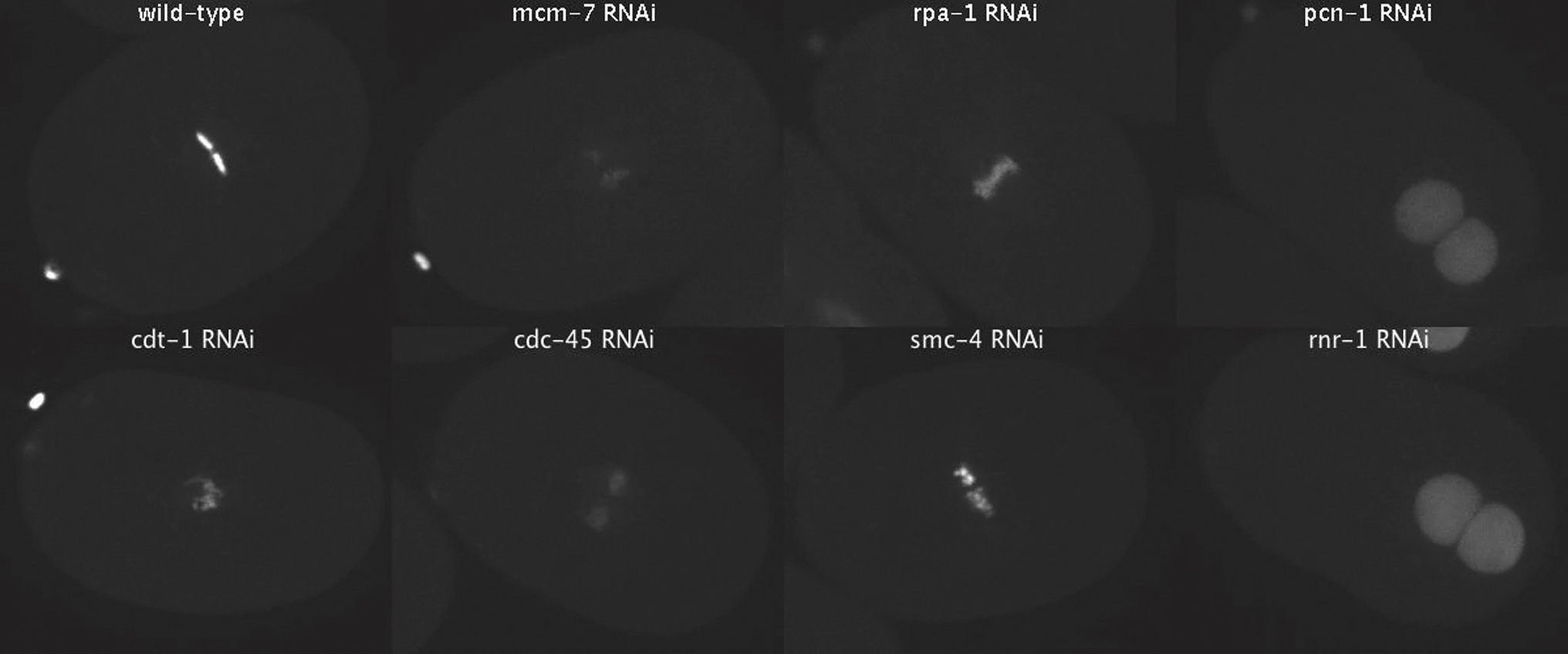

Supplement: Movie S3. Composite Video Showing Wild-Type, cdt-1 RNAi, mcm-7 RNAi, cdc-45 RNAi, rpa-1 RNAi, rnr-1 RNAi, and smc-4 RNAi Embryos Expressing GFP-Histone Progressing throughout the First Embryonic Cell Cycle, Related to Figure 2B [file mmc4.jpg]

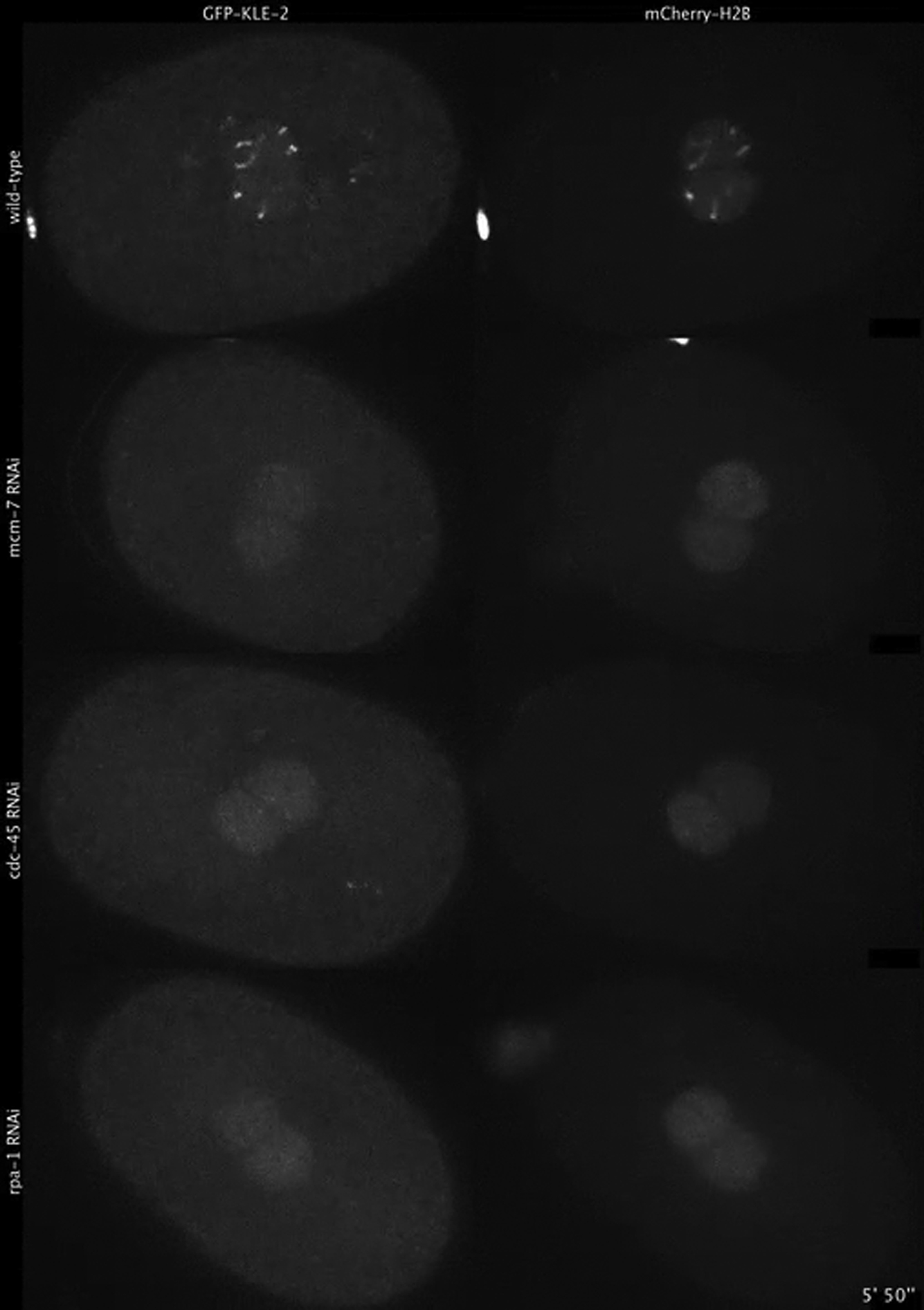

Supplement: Movie S4. Composite Video Showing, from Top to Bottom, Wild-Type, mcm-7, cdc-45, and rpa-1 RNAi Embryos Expressing GFP-KLE-2 and mCherry-Histone Progressing throughout Prophase of the First Embryonic Cell Cycle, Related to Figure 2D [file mmc5.jpg]

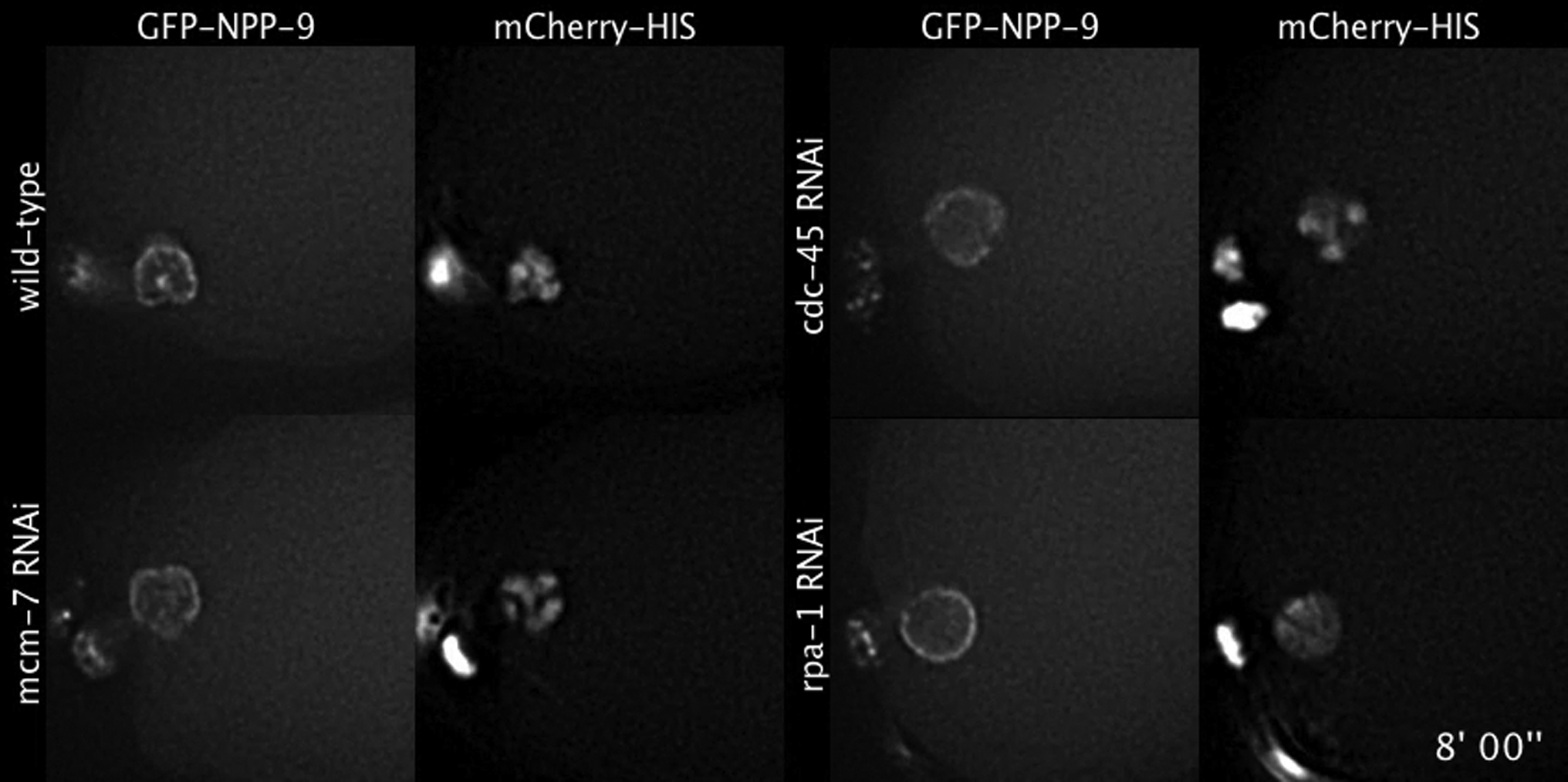

Supplement: Movie S5. Composite Video Showing, from Top to Bottom, Wild-Type, mcm-7 RNAi, cdc-45 RNAi, and rpa-1 RNAi Embryos Expressing GFP-NPP-9 and mCherry-Histone Progressing throughout the Second Meiotic Division and First Embryonic S Phase, Related to Figure 5A [file mmc6.jpg]

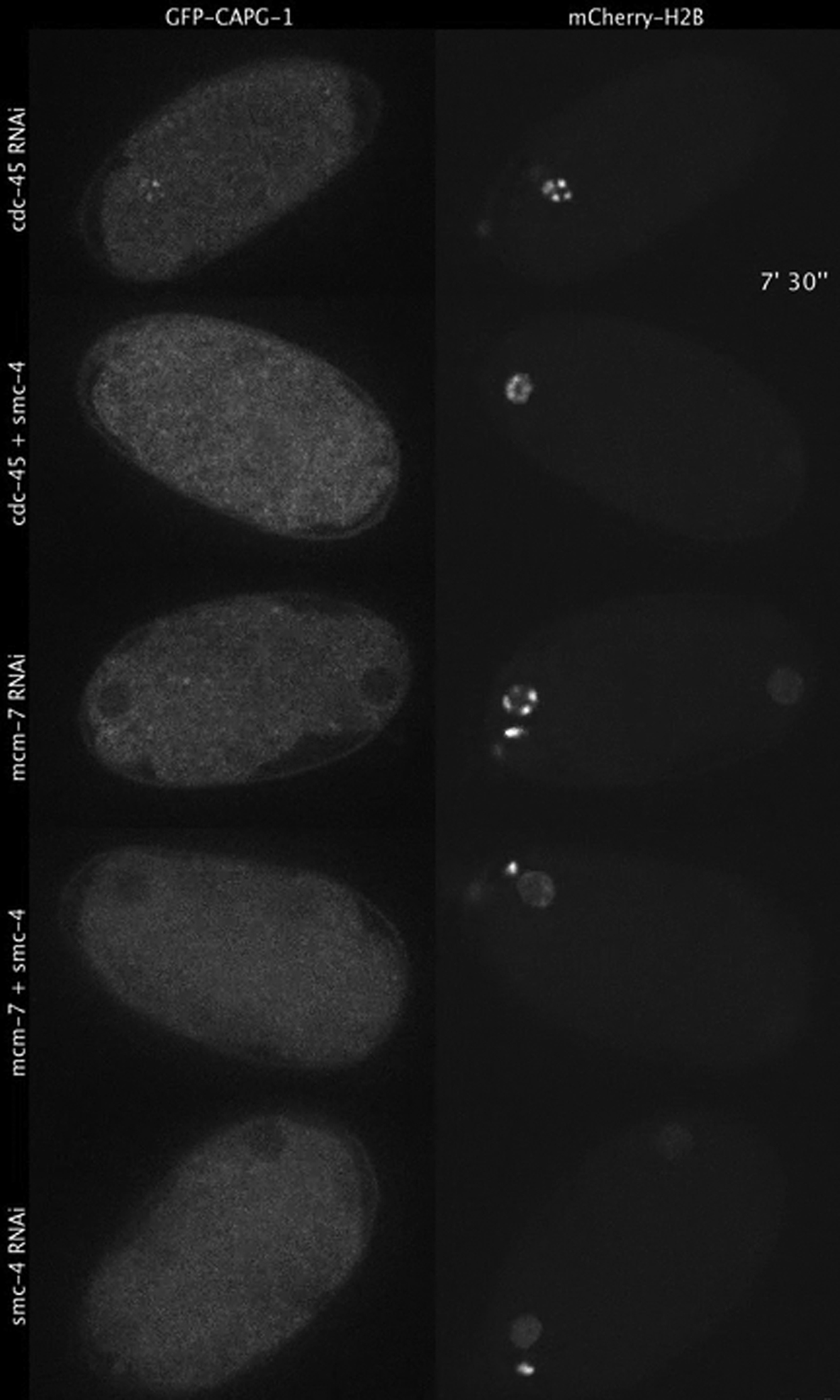

Supplement: Movie S6. Composite Video Showing, from Top to Bottom, cdc-45, Double cdc-45; smc-4, mcm-7, Double mcm-7; smc-4, and smc-4 RNAi Embryos Expressing GFP-CAPG-1 and mCherry-Histone Progressing throughout the Second Meiotic Division and First Embryonic Cell Cycle, Related to Figure 6B [file mmc7.jpg]

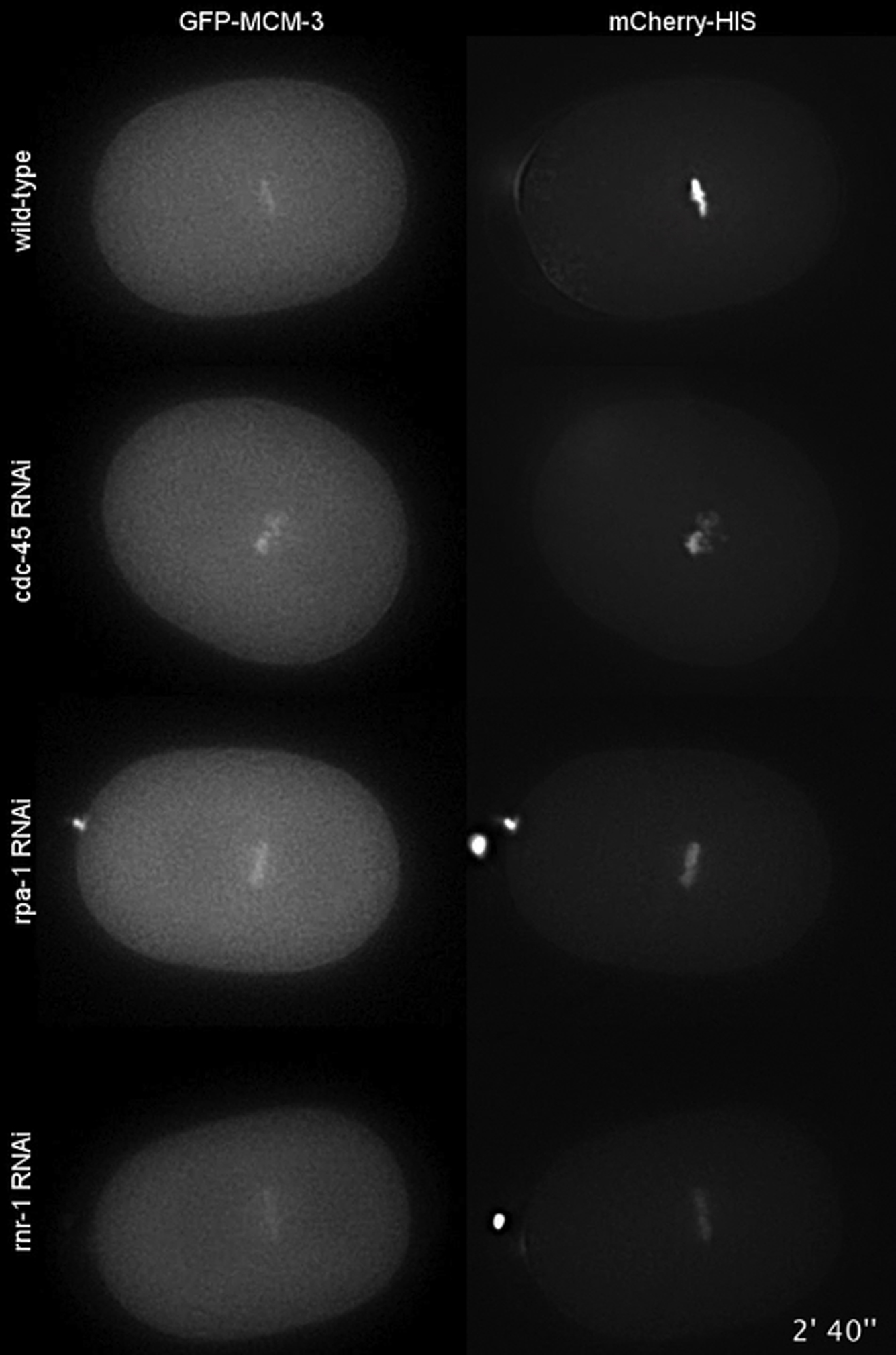

Supplement: Movie S7. Composite of Videos Showing, from Top to Bottom, Wild-Type, cdc-45 RNAi, rpa-1 RNAi, and rnr-1 RNAi Embryos Expressing GFP-MCM-3 and mCherry-Histone Progressing throughout the First Embryonic Mitosis, Related to Figure 7A [file mmc8.jpg]

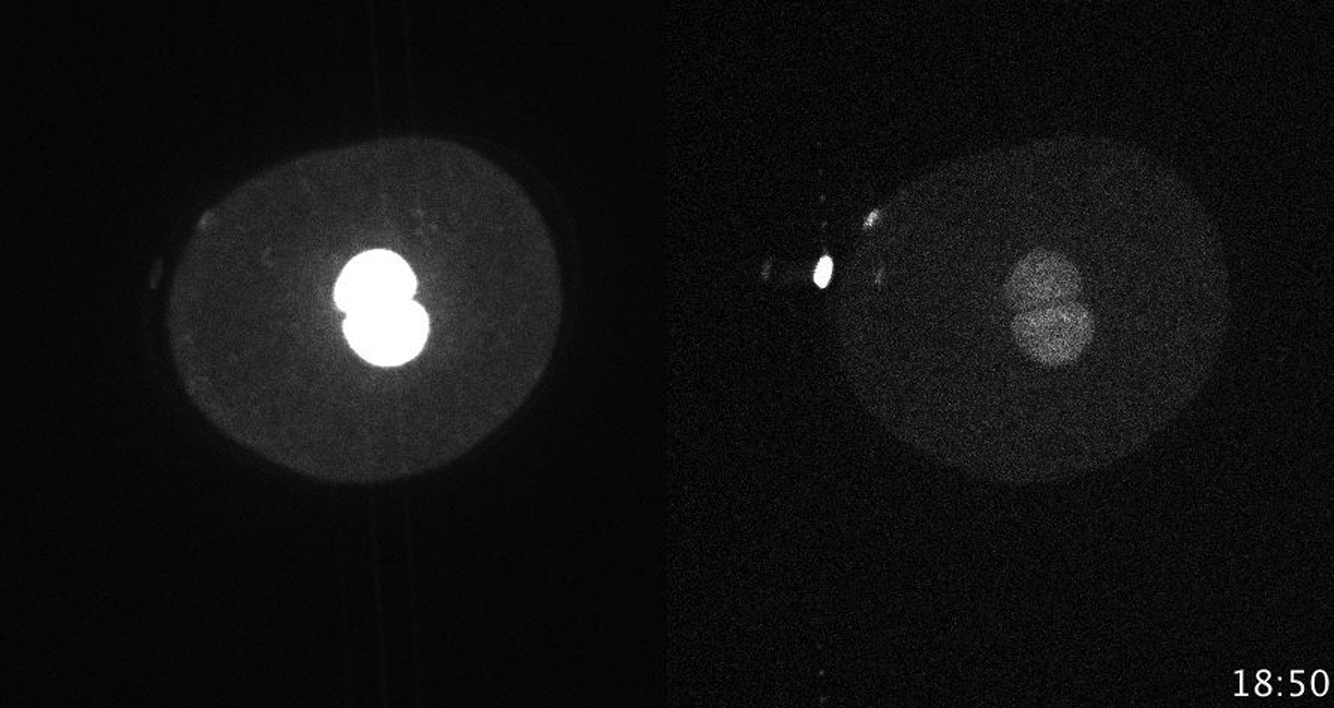

Supplement: Movie S8. Video of an Embryo Expressing GFP-MCM-3 and mCherry-Histone Progressing throughout the First Embryonic Cell Cycle, Related to Figure 7B — The female nuclei were photobleached during early S phase. [file mmc9.jpg]

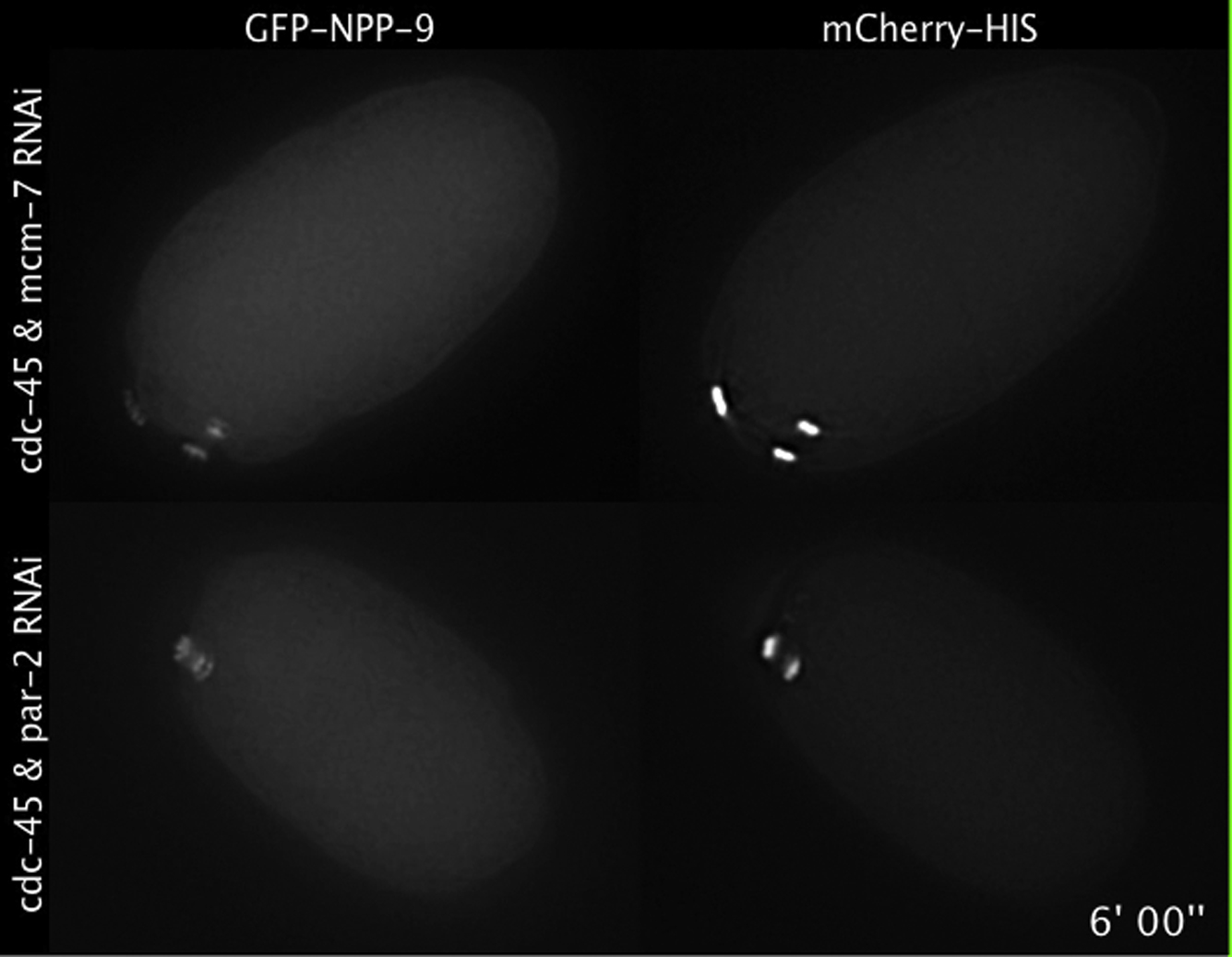

Supplement: Movie S9. Composite Video Showing Embryos Doubly Inactivated for cdc-45/par-2 and cdc-45/mcm-7, Related to Figure 7C — Embryos are expressing GFP-NPP-9 and mCherry-Histone progressing throughout the second meiotic division and first embryonic cell cycle. [file mmc10.jpg]

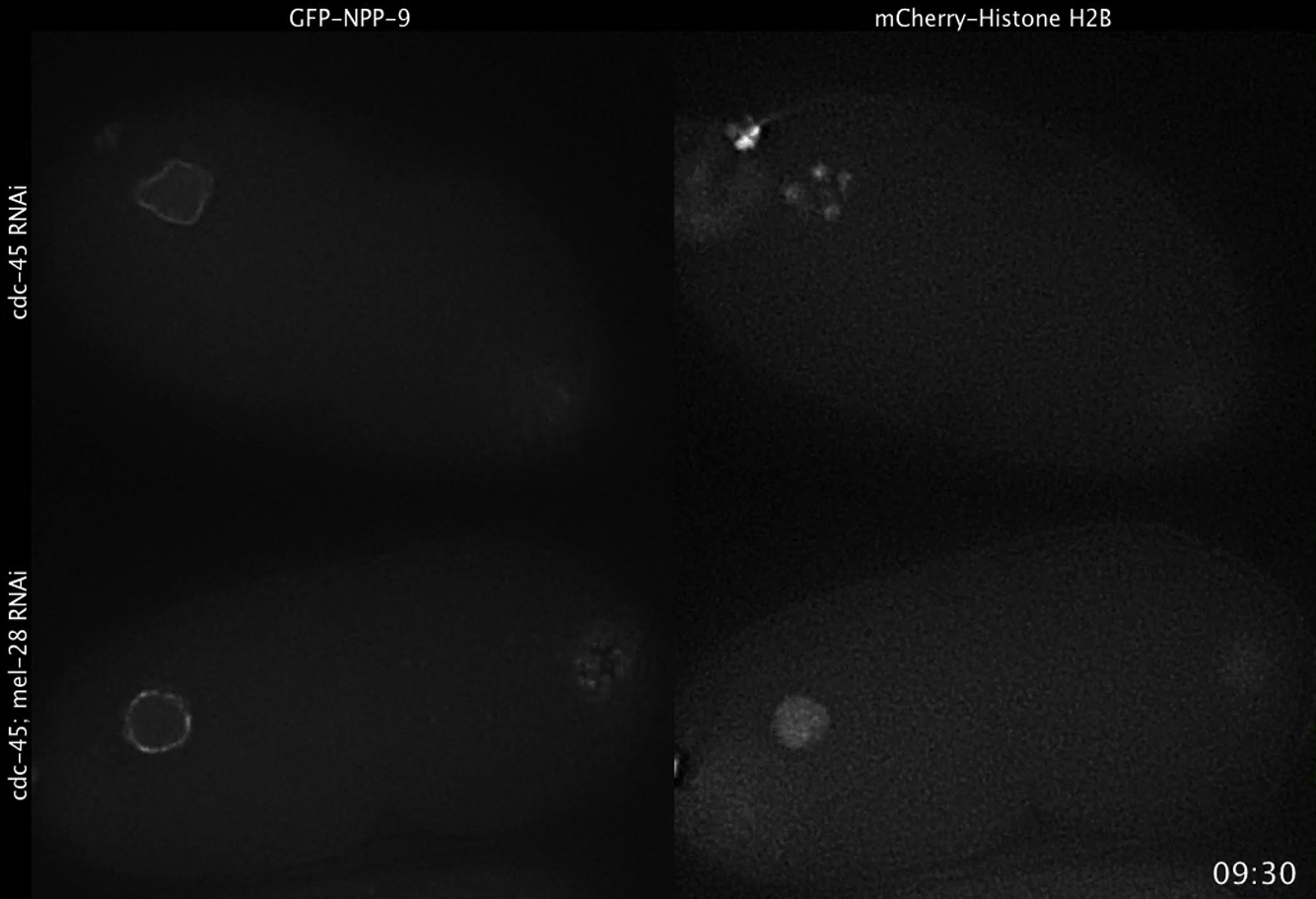

Supplement: Movie S10. Composite Video Showing cdc-45 and Double cdc-45; mel-28 RNAi Embryos Expressing GFP-NPP-9 and mCherry-Histone Progressing throughout the Second Meiotic Division and First Embryonic Cell Cycle, Related to Figure 7E [file mmc11.jpg]
